# The regulation loop of MARVELD1 interacting with PARP1 in DNA damage response maintains genome stability and promotes therapy resistance of cancer cells

**DOI:** 10.1038/s41418-023-01118-z

**Published:** 2023-02-07

**Authors:** Haoxiu Sun, Chao Liu, Fang Han, Xiaoyu Lin, Liangyu Cao, Chenxing Liu, Qiuyu Ji, Jinjin Cui, Yuanfei Yao, Bojun Wang, Yuanyu liao, Huan Nie, Yanqiao Zhang, Yu Li

**Affiliations:** 1 School of Life Science and Technology, Harbin Institute of Technology, Harbin, China; 2 Department of Gastrointestinal Medical Oncology, Harbin Medical University Cancer Hospital, Harbin, China; 3 Clinical Research Center for Colorectal Cancer in Heilongjiang, Harbin, China; 4 Key Laboratories of Tumor Immunology in Heilongjiang, Harbin, China; 5 Translational Medicine Research and Cooperation Center of Northern China, Heilongjiang Academy of Medical Sciences, Harbin, China

**Keywords:** Cancer, Cell biology

## Abstract

The DNA damage response (DDR) plays crucial roles in cancer prevention and therapy. Poly(ADP-ribose) polymerase 1 (PARP1) mediates multiple signal transduction in the DDR as a master regulator. Uncovering the regulatory factors of PARP1 contributes to a more comprehensive view of tumorigenesis and treatment strategies. Here, we reveal that MARVELD1 acts as a mediator of DDR to perform early events and maintain genome stability. Mechanistically, PARP1 PARylates MARVELD1 at D102, D118 and D130, and in turn, MARVELD1 stabilizes PARP1 by enhancing NAA50-mediated acetylation, thus forming a positive feedback loop. MARVELD1 knockout mice and their embryo fibroblasts exhibit genomic instability and shorter half-life of PARP1. Moreover, MARVELD1 partnering with PARP1 facilitates resistance to genotoxic drugs and disrupts PARP inhibitor (PARPi) effect in PDX model of colorectal cancer (CRC). Overall, our results underline the link between MARVELD1 and PARP1 in therapeutic resistance based on DDR and provide new insights for clinical tumor therapy of PARPi.

## Introduction

Currently, the 5-year survival prognosis of malignant tumors is mainly dependent on the clinical stage [1, 2]. Although targeted therapy, neoadjuvant radiotherapy and adjuvant chemotherapy are used globally as new therapeutic methods, the mortality and poor prognosis of higher-stage cancer have remained high [3]. Therapeutic resistance remains one of the deadlocks for poor prognosis [4, 5]. Among mechanism studies, the regulation of the DNA damage response (DDR) and maintenance of genomic stability in cancer cells have attracted extensive attention.

The factors maintaining genomic stability are barriers to cancer treatment and associated with poor prognosis, metastasis and therapeutic resistance [6]. It has clearly revealed that genomic stability is the key to improve the therapeutic resistance of cancer [7–9]. The DDR, as a complex protective system for cell survival, plays critical roles in maintaining genomic integrity and DNA repair. Thereby, DDR also is known as a negative effector of chemo/radiotherapy [6]. Targeting the DDR of cancer cells is becoming highlighted as a state-of-the-art strategy.

As a complex signal transduction network, the DDR is composed of multiple factors, involving in oxidation reduction, cell redox homeostasis, cell cycle arrest and regulation of cell death to repair DNA lesions [10]. In the DDR, a set of 450 genes encoding integral proteins has been described, and some mediators can recruit a “core” group of proteins, including specialized “sensor, transducer and effector” [11]. PARP1 is a key sensor of DNA damage. In order to signal the recruitment of DNA repair proteins to the site of DNA damage, one of the earliest events of DDR is the recruitment of PARP1 [12, 13]. PARP1 can promote the formation of a poly (ADP-ribose) polymer, termed PARylation, on its substrates as well as on itself [14, 15]. PARP1 function in the DDR relies heavily on posttranslational modifications (PTMs), such as PARylation, ubiquitination and acetylation [16]. However, the coordination of PARP1 PTMs in the cellular response remains incomplete. The pan-cancer analysis has shown that higher PARP1 expression is present in 27 tumor tissues [17]. PARP1 knockout or deletion cells are highly sensitive to genotoxic stress [18]. And PARP inhibitors (PARPi) targeting PARP1 have been widely studied for cancer treatment recently. Whereas the efficacy of PARPi is uncertain, and the reason is unclear.

This paper investigated the function of MARVELD1, a novel PARP1-interacting protein in DDR. MARVELD1 was defined as a DDR mediator to maintain genome stability and as a key indicator for poor prognosis. We found that PARylation of MARVELD1 depending on PARP1 was a crucial step for its nuclear translocation and formation of DDR network. MARVELD1 could stabilize PARP1 by enhancing NAA50-mediated acetylation. We verified that MARVELD1 depletion caused a higher genome instability and a shorter half-life of PARP1 using MARVELD1 knockout mice and their embryo fibroblasts (MEFs) by oxidants and chemo/radio-treatments. Moreover, partnership of MARVELD1 and PARP1 promotes resistance to DNA damaging therapy in colorectal cancer (CRC). These results reveal the mechanistic link between MARVELD1 and PARP1 in the regulation of therapeutic resistance, which can provide new insights for guidance of clinical tumor therapy based on PARPi.

## Results

### MARVELD1 regulates the genotoxic stress response of cancer cells and is associated with a poor prognosis

**Fig. 1:**
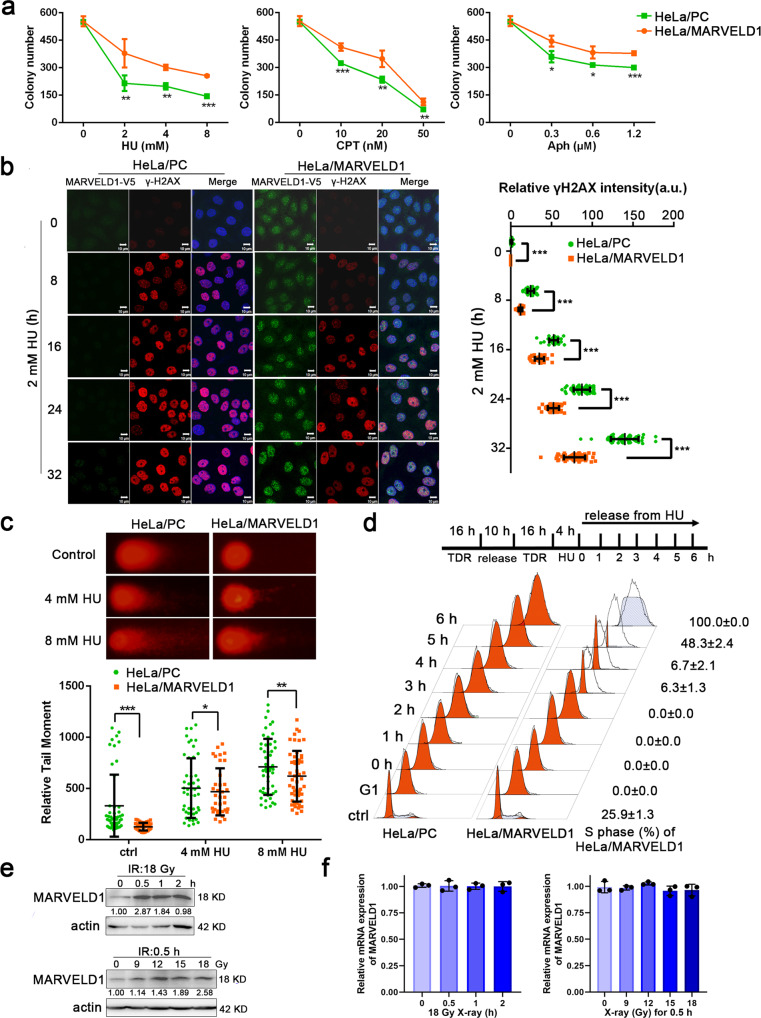
MARVELD1 regulates the genotoxic stress response of cancer cells and is associated with a poor prognosis. **a** The proliferation curves of HeLa/PC and HeLa/MARVELD1 cells treated by HU, CPT or Aph for 14 days. **b** Immunofluorescent (IF) staining and intensity analysis of MARVELD1-V5 and γ-H2AX in HU-treated HeLa/PC and HeLa/MARVELD1 cells. **c** Neutral comet assays of HeLa/PC and HeLa/MARVELD1 cells treated with HU for 4 h. **d** The cell cycle of HeLa/PC and HeLa/MARVELD1 cells were detected at different times after HU releasement. **e** The endogenous MARVELD1 protein level in HeLa cells was assessed after different times or dose treatment by X-ray radiation. **f** The endogenous MARVELD1 mRNA level in HeLa cells was assessed after different times or dose treatment by X-ray radiation. *p < 0.05, **p < 0.01, ***p < 0.001.

Our previous study illustrated that MARVELD1 exhibited significantly responsive changes in multiple tumor cells after exposure to stress-inducing stimuli [19]. Based on the finding, we explore the function of MARVELD1 in genotoxic stress. Cell survival assays showed that HeLa cells of stably expressing MARVELD1-V5 (HeLa/MARVELD1) had reduced cellular sensitivity to hydroxyurea (HU), camptothecin (CPT) and aphidicolin (Aph) compared to pcDNA3.1-transfected control cells (HeLa/PC) (Fig. 1a and Supplementary Fig. S1a, b), and HeLa/MARVELD1 showed fewer γH2AX foci (Fig. 1b) and shorter tail moment (Fig. 1c) after HU treatment. The results showed that the high level of MARVELD1 reduced the cell sensitivity to genotoxic stress and DNA damage. Then, cells were synchronized and treated with 4 mM HU, and all HeLa/MARVELD1 cells entered S phase at 6 h after release while HeLa/PC cells were blocked in the G1 phase (Fig. 1d, Supplementary Table S1, 2). And the γH2AX protein in synchronized HeLa/MARVELD1 cells was lower than that of HeLa/PC counterparts (Supplementary Fig. S1c). The results suggested MARVELD1 improves the replication restart following HU-induced stalling.

Next, we assessed endogenous MARVELD1 expression in HeLa cells under IR and UV treatment. Western blot (WB) analyses showed a marked MARVELD1 protein increase following 18 Gy X-ray treatment for 0.5 h and a dose-dependent induction of MARVELD1 at the 0.5 h time point (Fig. 1e). Whereas the indicated treatments did not affect MARVELD1 mRNA levels (Fig. 1f) Consistently, endogenous MARVELD1 exhibited responsive changes after UV radiation (Supplementary Fig. S1d, e). These findings indicate that endogenous MARVELD1 was upregulated in response to IR- or UV-induced DNA damage.

The high expression of genes regulating DDR is always associated with a poor prognosis in multiple tumors [20–23]. Therefore, we analyzed the TCGA database and found that the patients with high MARVELD1 expression performed poorer overall survival among nine tumor types (including LGG, LUAD, STAD, GBM, ACC, BRCA, BLCA, LIHC and COAD) (Supplementary Fig. S1f).

The data prove that MARVELD1 enhances the tolerance of cells to genotoxic stimuli and associates with prognosis.

### MARVELD1 is a mediator of DDR network and interacts with PARP1

**Fig. 2:**
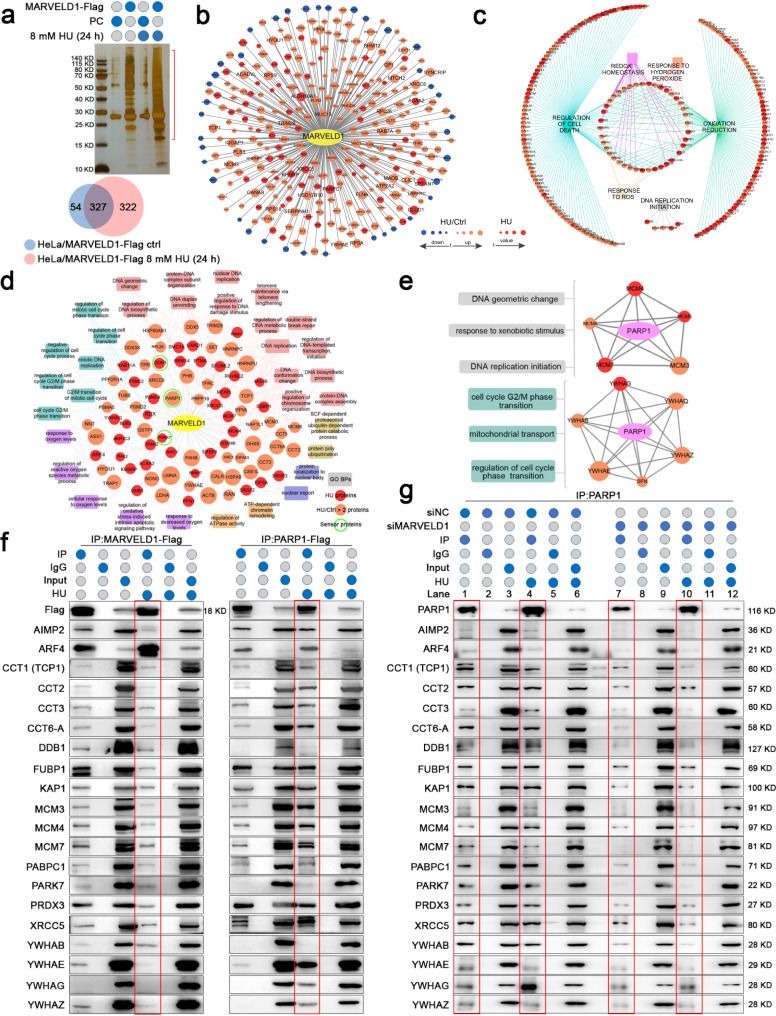
MARVELD1 interacts with DDR proteins and is a DDR mediator. **a** Lysates of HU treatment HeLa/PC or HeLa/MARVELD1-Flag cells were immunoprecipitated with anti-Flag antibody. The gel pieces containing regions of interest (area shown in the bracket) were analyzed by mass spectrometry. Venn diagram showing the number of MARVELD1 interaction proteins. **b** The difference of protein–protein interaction (PPI) was obtained between the control and HU group. **c** GO analysis were performed for the MARVELD1-interacting proteins. **d** DDR-related GO terms and their correlated MARVELD1 interactors were constructed into a DDR-related function-protein network (the green circles mark the sensor proteins). The dot size indicates more unique peptides or a higher ratio of HU/Ctrl. **e** Schematic diagram show that MARVELD1 and PARP1 share interacting protein families of MCM and YWHA (right), along with their related functions (left). **f** MARVELD1 and PARP1 interactors were confirmed by Co-immunoprecipitation (Co-IP) and Western blotting (WB) in HeLa cells (the red squares mark the interactions after HU treatment). **g** MARVELD1 knockdown whether affect the interaction between PARP1 and DDR proteins was confirmed by Co-IP and WB in HeLa cells (the red squares mark the interactions).

To reveal the regulatory mechanism of MARVELD1 in genotoxic stress response, we explored the protein interactors after HU treatment. The HeLa cell lysates were resolved by SDS–PAGE, followed by silver staining and mass spectrometry. The data showed that the interacting proteins of MARVELD1 were increased remarkably under HU stress, 649 proteins in the HU-treated group and 381 proteins in the control group (Fig. 2a, b). And the MARVELD1 interactors of HU treated group were highly enriched in the DDR system, including the regulation proteins of cell death, redox homeostasis, oxidation reduction and DNA replication initiation (Fig. 2c). A large number of DDR effectors and specialized sensors (e.g., PARP1, PARK7 and DDB1) of detecting DNA damage were obtained (Fig. 2d). There were also many multifunctional protein families among the interactors of MARVELD1, such as the MCM complex proteins (the initiation of DNA replication and regulation of cell cycle) and YWHA family (also called 14-3-3 proteins, mediating signal transduction). More importantly, these proteins also interacted with PARP1, a sensor protein of DDR and regulation of cell death (Fig. 2e). Thus, some DDR proteins interacting with MARVELD1 and PARP1 were confirmed by Co-IP (Fig. 2f). MARVELD1 knockdown (Supplementary Fig. S2a) reduced the interactions between PARP1 and the DDR proteins (Fig. 2g, compare lane 1 with 7; 4 with 10). These results indicate that MARVELD1 could participate in early DDR processing as a mediator and interact with PARP1, and might have the ability to recruit sensor and effector proteins.

**Fig. 3:**
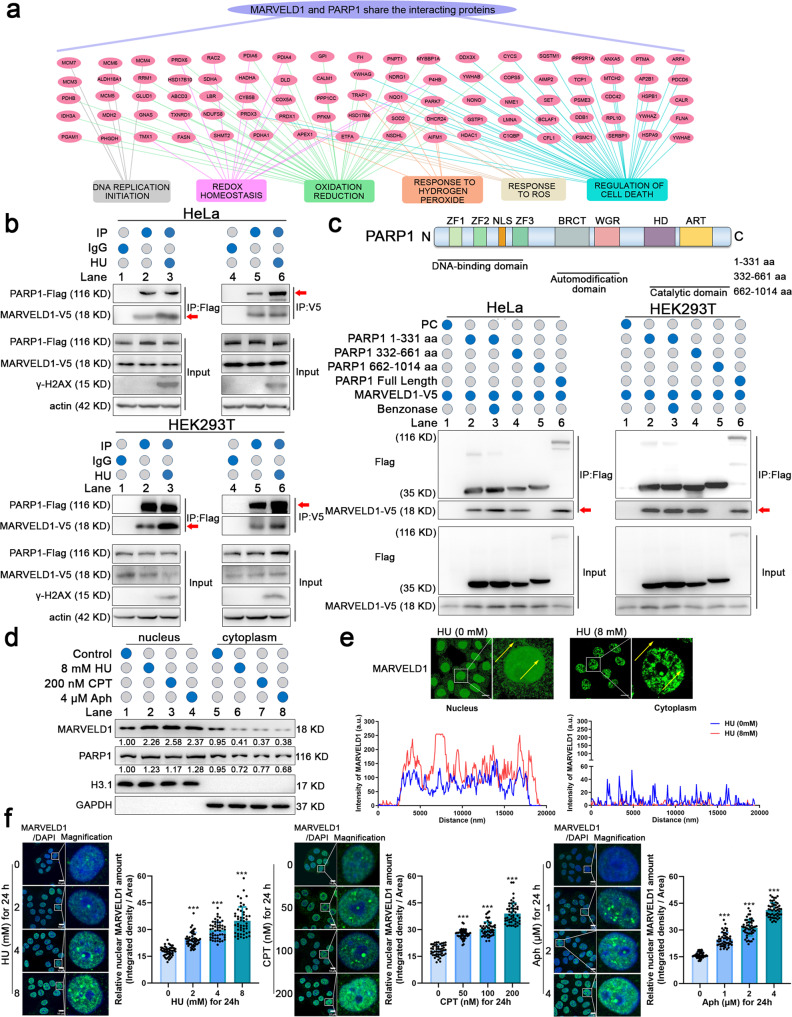
MARVELD1 binds with PARP1 and clusters in the nucleus during HU stress. **a** PPI were conducted from mass spectrometry data (Fig. 2) by the common interactors of PARP1 and MARVELD1. **b** The interaction of MARVELD1 and PARP1 was analyzed by IP with IgG or the indicated antibody after treated with 8 mM HU (HeLa cells) or 4 mM HU (HEK293T cells) for 24 h, followed by WB. **c** Schematic diagram showing the domains of PARP1. ZF zinc finger, NLS nuclear localization signal, BRCT BRCA1 C-terminus, WGR Trp-Gly-Arg domain, HD helical subdomain, ART ADP-ribosyl transferase. Truncated PARP1-flag and V5-tagged MARVELD1 were co-transfected into HeLa and HEK293T cells. Lysates were analyzed by IP of Flag with a subsequent WB. **d** Nuclear and cytoplasmic protein extraction assays of HeLa cells with the indicated treatment for 24 h were conducted, followed by WB. **e** IF staining of HeLa cells was used to determine the nucleus translocation of MARVELD1 after HU treatment, and the line graphs represent the fluorescence intensity of MARVELD1 at the arrow by ZEN software. Scale bar: 10 μm. **f** MARVELD1 clustered in nucleus was analyzed by IF staining when HeLa cells were treated by different dose of HU, CPT or Aph. Green staining indicates MARVELD1, blue staining indicates DAPI. The relative nuclear MARVELD1 amount (integrated density/area) per cell was quantified by ImageJ and 50 cells were measured per treatment. Scale bar: 10 μm. ***p < 0.001.

PARP1 is a key enzyme with multiple functions and involved in DDR and cell survival [12, 24]. Hence, we analyzed the interacted peptides of MARVELD1 and PARP1 (Supplementary Fig. S2b, c). And the proteins interacted both with MARVELD1 and PARP1 were showed by protein–protein interaction (PPI) network (Fig. 3a), involving in DNA replication initiation, regulation of cell death, oxidative stress response and cell redox homeostasis. Moreover, we proved that the interaction between MARVELD1 and PARP1 was greatly enhanced following exposure to HU in HeLa and HEK293T cells (Fig. 3b and Supplementary Fig. S2d).

To define the domain of PARP1 that mediates its interaction with MARVELD1, we generated three PARP1 truncations based on the known functional domains (N-terminal DNA-binding domain, automodification domain, and C-terminal catalytic domain) [25]. Co-IP assays showed that the DNA-binding domain or automodification domain could be responsible for interacting with MARVELD1 (Fig. 3c, indicated by the arrows). And the interaction between the PARP1 DNA-binding domain and MARVELD1 was not mediated by DNA, as the interaction was maintained in the presence of benzonase which degrades all forms of DNA and RNA (shown in Fig. 3c Lane 3). Then, subcellular fraction assays of HeLa cells indicated that both MARVELD1 and PARP1 were translocated to the nucleus during genotoxic stress (Fig. 3d). Immunofluorescent (IF) staining results showed that more MARVELD1 and PARP1 were colocalized in HeLa cell nuclei (Supplementary Fig. S2e), and MARVELD1 formatted foci in response to HU treatment (Fig. 3e). In addition, the nucleus translocation induced by HU, CPT or Aph showed a dose-dependent manner (Fig. 3f and Supplementary Fig. S2f). Of interest, clustered MARVELD1 in the nucleus appeared clearly as spots with the dose of the treatment increased, which was accompanied by more serious DNA damage. The WB results showed a nearly 2.5-fold increase in nuclear MARVELD1 levels and cytoplasmic MARVELD1 was 0.4 times of the original level (Fig. 3d), whereas the total protein level increased slightly (Supplementary Fig. S2g). Thus, the increase of nuclear MARVELD1 in DNA damage mainly dependent on the nucleus translocation. These data revealed that MARVELD1 interacts with PARP1 in nucleus and that PARP1 is a noteworthy interacting partner of MARVELD1 under HU treatment.

### MARVELD1 is PARylated and clustered in the nucleus depending on PARP1 upon DDR

**Fig. 4:**
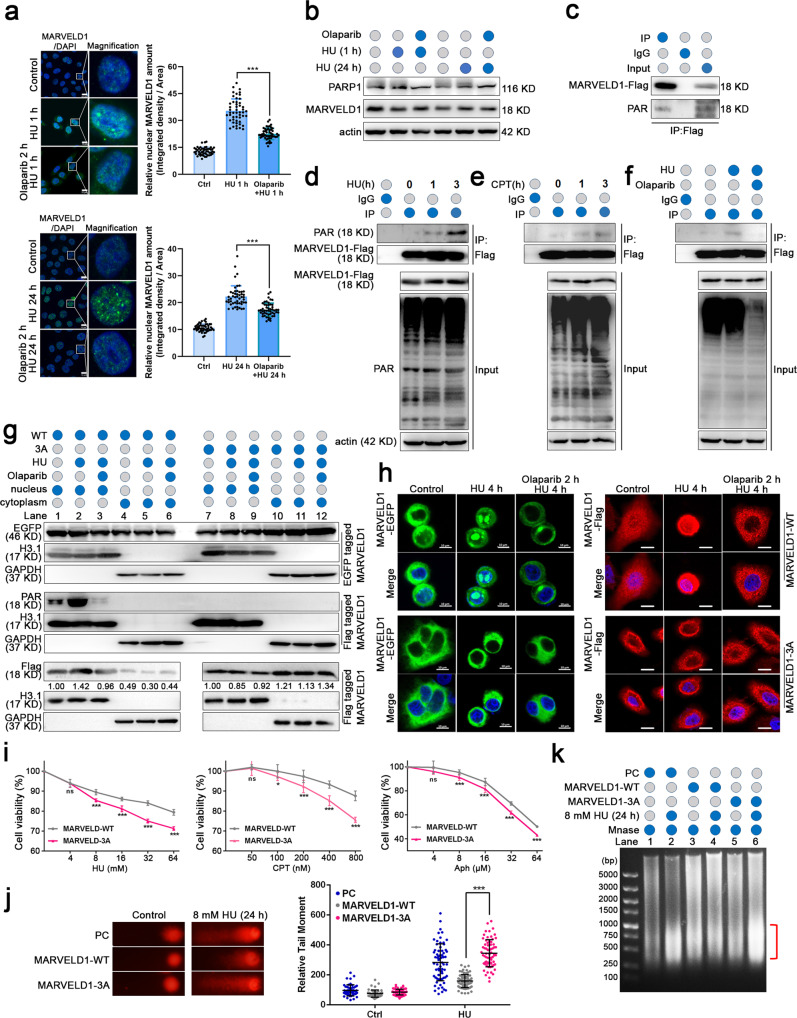
MARVELD1 is modified by PARP1 and clustered in the nucleus dependent on its PARylation under DDR. **a** The MARVELD1 subcellular location was detected by IF staining in HeLa cells treated with olaparib or HU. Green staining indicates MARVELD1, blue staining indicates DAPI. The relative nuclear MARVELD1 amount (integrated density/area) per cell was quantified by ImageJ and 50 cells were measured per treatment. Scale bar: 10 μm. **b** The MARVELD1 and PARP1 protein levels were detected by WB in HeLa cells with the indicated treatments. **c** The PARylation of MARVELD1 was analyzed in HeLa/MARVELD1-Flag cells by IP with IgG or anti-Flag antibody, followed by WB with the PAR antibodies. **d**, **e** HeLa cells were treated with 4 mM HU or 0.5 μM CPT for the indicated times and then subjected to IP to explore the PARylation adjustment. **f** The MARVELD1 PARylation was analyzed in HeLa cells pretreated with 5 μM olaparib for 1 h and then treated with 4 mM HU for another 1 h. Lysates were analyzed by IP. **g** Nuclear and cytoplasmic protein extraction assays of HeLa/MARVELD1-WT or -3A were conducted, followed by WB. **h** The location change of MARVELD1 was detected by IF staining of HeLa/MARVELD1-EGFP and HeLa/MARVELD1-Flag cells after olaparib treatment. Scale bar: 10 μm. **i** Cell viability assays of HeLa/MARVELD1-WT or -3A to verify the cellular sensitivity in response to increasing doses of HU, CPT and Aph for 24 h. **j** Neutral comet assays of HeLa/PC, HeLa/MARVELD1-WT and HeLa/MARVELD1-3A cells treated with HU. Representative images and quantified tail moments are shown for each group. **k** Isolated nuclei of HeLa/PC, HeLa/MARVELD1-WT and HeLa/MARVELD1-3A treated with HU were subjected to MNase assays. *p < 0.05, **p < 0.01, ***p < 0.001.

As already reported, the major functions of PARP1 in the DDR depend on PARylation activity [12]. Thus, we determined whether MARVELD1 nuclear translocation was associated with PARylation. HeLa and SiHa cells were pretreated with the PARP inhibitor olaparib for 2 h and then subjected to HU treatment. Olaparib could block MARVELD1 translocation to the nucleus (Fig. 4a and Supplementary Fig. S3a), but showed no effect on the MARVELD1 protein amount (Fig. 4b). We also sought PARylation-related molecules among MARVELD1 interactors. As shown in Supplementary Fig. S3b, PABPC1 and ZC3HAV1 may be ARBDs (ADP-ribose readers), IDH3a and NQO1 as PARP feeders and NA(P)D^+^-related regulatory proteins, and PARP1 should be an ADP-ribose writer [24]. For this analysis, we justified the PARylation state of MARVELD1 catalyzed by PARP1 using anti-poly (ADP-ribose) (PAR)polymer antibody (Fig. 4c). MARVELD1 PARylation was enhanced by treatment with HU (Fig. 4d) or CPT (Fig. 4e), while the PARylation was reduced by treatment with olaparib (an inhibitor of PARP1) (Fig. 4f). Meanwhile, the PARG inhibitor PDD00017273, which blocked the degradation of the poly (ADP-ribose) chain [26, 27], or olaparib was used to induce or reduce the PARylation level of HeLa cells. The data showed that endogenous MARVELD1 protein and half-life were not affected by PARylation modification (Supplementary Fig. S3c, d). These results indirectly demonstrate that the nuclear translocation of MARVELD1 in DDR depends on the PARylation by PARP1.

PARylation most commonly takes place on aspartate (D), glutamate (E) and lysine (K) residues of target proteins [26]. Sequence alignments of MARVELD1 showed nine residues (K53, K83, E85, D102, D118, K129, D130, K169 and E171) may be potentially PARylated. Therefore, we substituted the nine residues with alanine (A) individually, transfected these recombinant mutants into HeLa or HEK293T cells. We found that mutation of D102, D118 or D130 triggered a significant decrease in MARVELD1 PARylation compared with its WT counterpart and other single mutants after HU treatment (Supplementary Fig. S3e). Then, we created a combination mutant in which D102, D118, and D130 were all substituted with alanine (A) (termed 3A) and analyzed its PARylation modification. The 3A mutant showed no detectable PARylation after HU treatment (Supplementary Fig. S3f). Furthermore, the subcellular localization of MARVELD1-3A revealed markedly altered its location, and reduced its translocation to the nucleus after HU treatment (Fig. 4g,h). The cell viability assays further verified that expression of PARylation-defective MARVELD1 led to enhanced cellular sensitivity to genotoxic stress (HU, CPT and Aph) in HeLa cells (Fig. 4i). And cells with MARVELD1-3A showed a longer relative tail moment, implying stronger DNA damage (Fig. 4j). Consistently, the MNase sensitivity assay showed that HeLa/MARVELD1-3A had more broken DNA than HeLa/MARVELD1 group after HU stress (Fig. 4k). These results reveal that PARylation of MARVELD1 is dependent on PARP1 activity and is important for maintaining genome integrity in the face of DNA damage.

### MARVELD1 enhances PARP1 stability by promoting NAA50-dependent acetylation

**Fig. 5:**
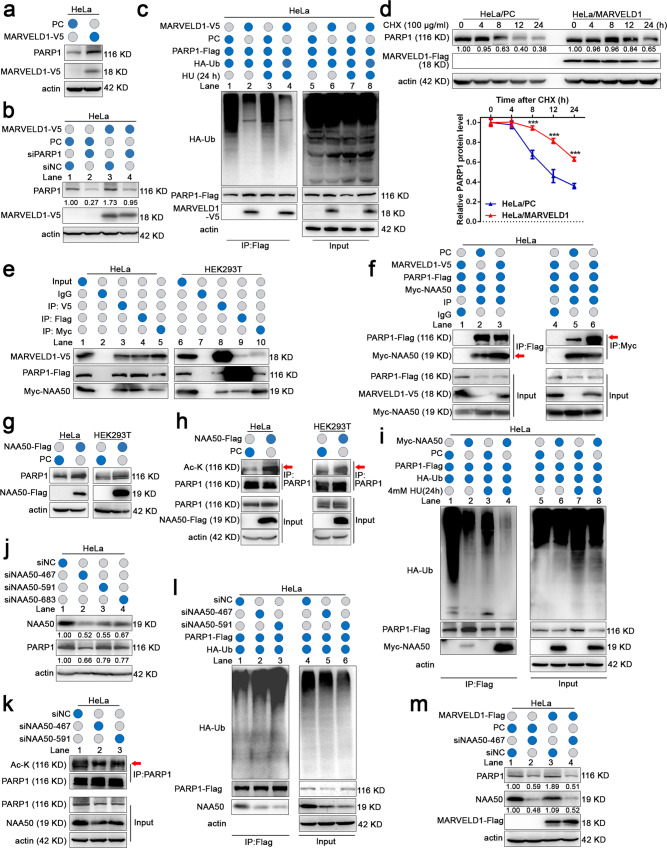
MARVELD1 enhances PARP1 stability by promoting NAA50-dependent acetylation. **a** PARP1 protein level in HeLa/MARVELD1 cells. **b** PARP1 protein level was analyzed by WB when MARVELD1-V5 and PARP1 siRNA was co-transfected in HeLa cells after 48 h. **c** The Ubiqutin-PARP1 was analyzed in HeLa cells. After cells treated with or without 4 mM HU for 24 h, MG-132 was added for 6 h, and lysates were subjected to IP and WB analysis. **d** The half-life of PARP1 was detected by WB in HeLa cells treated with CHX for the indicated time points. Relative PARP1 protein levels(PARP1/actin) were quantified and plotted in the lower panels. **p < 0.01, ***p < 0.001. **e** The interaction of MARVELD1, PARP1 and NAA50 was explored by Co-IP in HeLa and HEK293T cells transfected with the indicated recombinants after 48 h. **f** The effect of MARVELD1 on the interaction of PARP1 and NAA50 was tested by IP at 48 h after HeLa cells were transfected. **g**, **h** PARP1 protein or acetylation levels were examined in HeLa/NAA50-Flag or HEK293T/NAA50-Flag cells. The immunoprecipitated PARP1 was adjusted to equal levels to make the levels of acetylated PARP1 comparable to those of immunoprecipitated PARP1. **i** The Ubiqutin-PARP1 level was checked in HeLa/NAA50-Flag cells treated with or without 4 mM HU for 24 h. After HU treatment, cells were treated with MG-132 for 6 h, and lysates were subjected to IP and WB analysis. **j**, **k** PARP1 protein and acetylation level was detected in HeLa/ siNAA50 cells after 48 h transfection. **l** The Ubiqutin-PARP1 level was verified in HeLa/siNAA50 cells. At 48 h transfection, HeLa cells were treated with MG-132 for 6 h, and lysates were subjected to IP and WB analysis. **m** PARP1 protein level was analyzed in HeLa/MARVELD1 cells transfected with the NAA50 siRNA. WB was performed after 48 h transfection.

In view of the interaction between MARVELD1 and PARP1 in the DDR, we examined the effect of MARVELD1 on PARP1. The above data exhibited MARVELD1 knockdown could decrease PARP1 protein level (Supplementary Fig. S2a). After confirming that overexpression of MARVELD1 could increase PARP1 expression (Fig. 5a and Supplementary Fig. S4a), we detected that overexpression of MARVELD1 restored siRNA-induced downregulation of PARP1 protein in HeLa and HEK293T cells (Fig. 5b and Supplementary Fig. S4b), but not PARP1 mRNA (data not shown). Conversely, the level of MARVELD1 protein was not apparently affected by PARP1 knockdown (Supplementary Fig. S4c). As PARP1 stability controlled by ubiquitin-mediated proteolysis is a key feature in DDR and DNA repair [28, 29], we addressed that MARVELD1 affect the ubiquitination and degradation of PARP1. We observed that MARVELD1 markedly decreased the ubiquitination levels of PARP1-Flag (Fig. 5c and Supplementary Fig. S4d). And the half-lives of endogenous PARP1 protein were prolonged remarkably in HeLa/MARVELD1 or HEK293T/MARVELD1 cells (Fig. 5d and Supplementary Fig. S4e).

Considering deacetylase inhibitors can enhance protein levels of PARP1 [30] through proving acetylation and inhibiting ubiquitination. Our attention further was drawn to histone acetyltransferase NAA50, which is a potential partner of MARVELD1 from our proteomic assays (Supplementary Fig. S4f). Reciprocal IP assays showed that MARVELD1, PARP1 and NAA50 formed a ternary complex in HeLa and HEK293T cells (Fig. 5e), and NAA50 bound to the central region of PARP1 (Fig. S4g, red triangles). Exogenously expressed MARVELD1-V5 improved the interaction of PARP1-Flag and Myc-NAA50 (Fig. 5f and Supplementary Fig. S4h, red arrows).

Moreover, we ectopically expressed NAA50-Flag and found that the endogenous PARP1 expression (Fig. 5g) and acetylation were upregulated (Fig. 5h, red arrows). Overexpression of NAA50 then suppressed PARP1 ubiquitination independent of HU treatment (Fig. 5i and Supplementary Fig. S4i). On the other hand, when NAA50 expression was knockdown by siRNA (Fig. 5j and Supplementary Fig. S4j), the level of PARP1 acetylation was decreased (Fig. 5k and Supplementary Fig. S4k, red arrows). And NAA50 knockdown also promoted PARP1 ubiquitination (Fig. 5l and Supplementary Fig. S4l). Then, we explicated the relation among MARVELD1, NAA50 and PARP1. MARVELD1-Flag was ectopically expressed alone or in combination with a siRNA against NAA50. NAA50 knockdown could block the upregulation of PARP1 induced by MARVELD1 (Fig. 5m and Supplementary Fig. S4m). The above results clearly support that MARVELD1 enhances PARP1 acetylation depending on NAA50, and promotes PARP1 stability.

### MARVELD1 depletion impairs the genome stability and the levels of PARP1 protein in mice

**Fig. 6:**
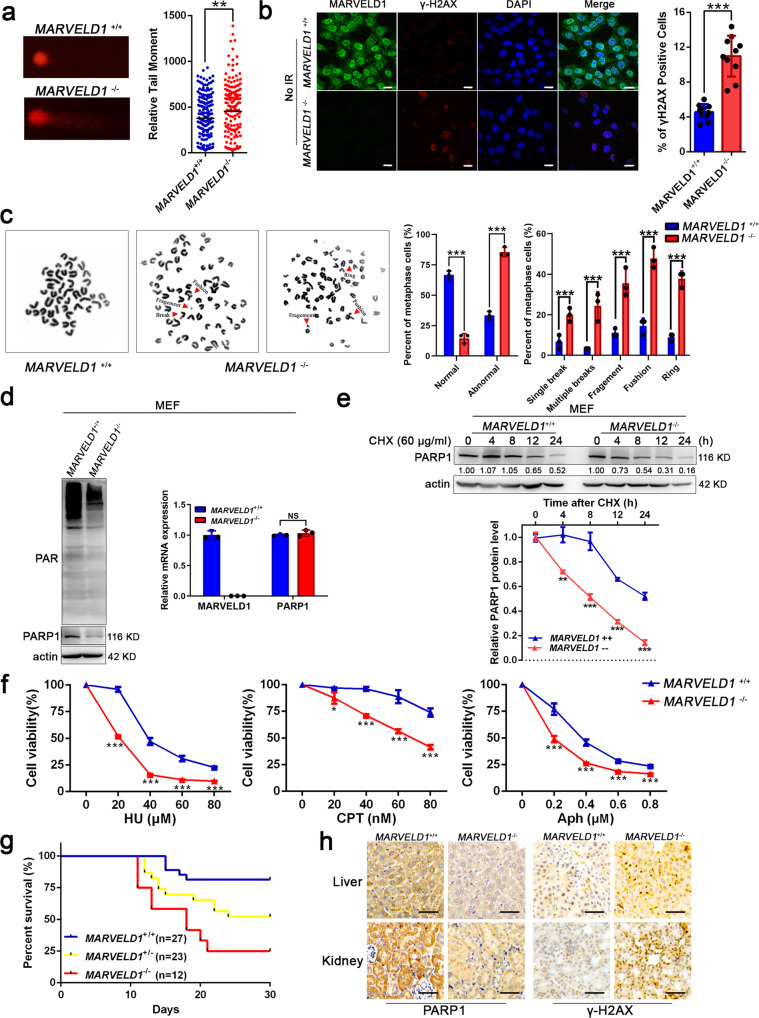
MARVELD1 depletion impairs the genome stability and the levels of PARP1 protein in mice. **a** Neutral comet assays of *MARVELD1*^+/+^ and *MARVELD1*^−/−^ MEFs. Representative images and quantified tail moments are shown for each group. **b** γ-H2AX foci of untreated MEFs are shown. Scale bar: 20 μm. **c** Metaphase spreads in untreated MEFs were performed to analyze chromosome aberrations. Arrows indicate chromosome aberrations. *n* ≥ 100 metaphase cells in per experiment. **d** WB analysis of the endogenous levels of the PAR chain and PARP1 protein in MEFs (left). The quantitative data (right) of mRNA levels are from three independent experiments. **e** The half-life of PARP1 in MEFs was detected when cells were treated with CHX and analyzed by WB. Relative PARP1 protein levels (PARP1/actin) were quantified and plotted in the lower panels. **f** Cell viability assays of MEFs in response to increasing doses of HU, CPT and Aph at 48 h were performed. **g** Five- to eight-week-old *MARVELD1*^+/+^ (*n* = 27), *MARVELD1*^+/−^ (*n* = 23) and *MARVELD1*^−/−^ (*n* = 12) littermates were subjected to 5 Gy X-ray radiation of the whole body and monitored for 30 days. **h** The levels of endogenous PARP1 and DNA damage were measured by immunohistochemistry (IHC) of liver and kidney tissues in mice treated as in g. Scale bar: 50 μm. *p < 0.05, **p < 0.01, ***p < 0.001.

Moreover, MARVELD1 knockout (*MARVELD1*^*−/−*^) mice of the C57BL/6 J strain [31] and its embryo fibroblasts (MEFs) (Supplementary Fig. S5a, b) were used to address the function of MARVELD1 in vivo. *MARVELD1*^−/−^ MEFs showed more spontaneous DNA damage with a longer relative tail moment (Fig. 6a), more γH2AX foci (Fig. 6b) and a nearly 4-fold increase in chromosomal aberrations compared to wild-type (*MARVELD1*^+/+^) MEFs (Fig. 6c). These results suggested an intrinsic defect in genome stability of *MARVELD1*^−/−^ cells. We also noticed that PARP1 protein levels and PARylation activity (PAR bands) were impaired in *MARVELD1*^−/−^MEFs, but the mRNA was unchanged (Fig. 6d). Meanwhile, the half-life of endogenous PARP1 protein was markedly shortened in *MARVELD1*^−/−^MEFs (Fig. 6e). When exposed to HU, CPT and Aph for 48 h, the viability of *MARVELD1*^−/−^ MEFs was more hypersensitive than that of *MARVELD1*^+/+^MEFs (Fig. 6f). And PARP1 was downregulated in *MARVELD1*^−/−^ cells whereas another sensor protein DDB1 was upregulated, which might be a novel form of DDR activation with PARP1 degradation (Supplementary Fig. S5c).

Increased radiation sensitivity is a hallmark of a defective DDR [32]. As shown in Fig. 6g, 25% of *MARVELD1*^−/−^mice were alive 30 days after radiation with a dose of 5 Gy, while 80% of *MARVELD1*^+/+^ and 50% of *MARVELD1*^+/-^ mice were still alive. Immunochemical assays indicated that the livers and kidneys of *MARVELD1*^−/−^ mice exhibited more γ-H2AX signals and lower levels of endogenous PARP1 after radiation (Fig. 6h), while 8-OHdG, another marker of DNA damage, also showed significant difference (Supplementary Fig. S5d). In addition, to verify the regulatory role of MARVELD1 in eliminating oxidative DNA damage, *MARVELD1*^+/+^, *MARVELD1*^+/−^ and *MARVELD1*^−/−^ mice were administered 1500 ppm KBrO3 in drinking water for 5 weeks. The ELISA data of 8-OHdG in blood serum, brain and liver tissues of MARVELD1^−/−^ mouse grew higher compared to the wild-type (Supplementary Fig. S5e). The results of IF staining in brain and liver tissues were consistent with the data of ELISA (Supplementary Fig. S5f).

These data from MARVELD1 depletion mice demonstrate that MARVELD1 plays important roles in regulating the DDR induced by genotoxic stress, and maintaining genome stability.

### The partnership of MARVELD1 and PARP1 induces resistance to 5-FU combining with olaparib in CRC

**Fig. 7:**
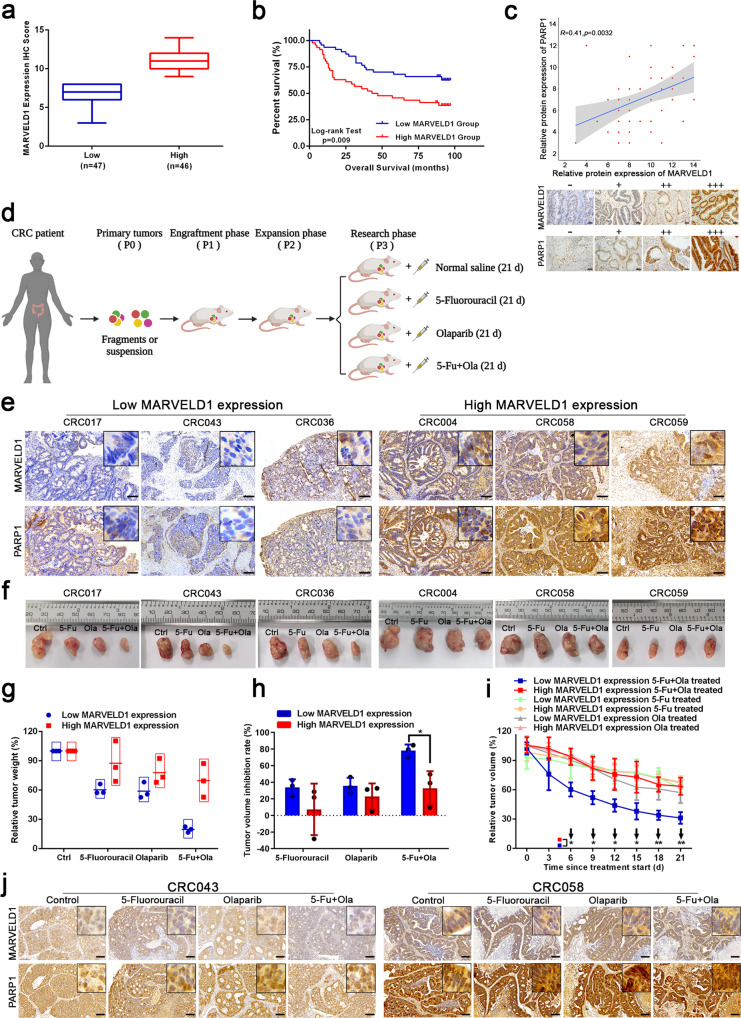
The partnership of MARVELD1 and PARP1 induces resistance to 5-FU and olaparib in CRC. **a** According to the IHC score, the patients were divided into a low MARVELD1 expression group (*n* = 47) and a high MARVELD1 expression group (*n* = 46). **b** The OS curves for the two groups in the CRC tissue microarray. **c** The correlation between PARP1 and MARVELD1 expression in the CRC tissue microarray according to the IHC score and the representative images of MARVELD1 and PARP1 protein expression in a CRC tissue microarray from 93 patients (−, +, ++, +++). **d** Schematic diagram showed the PDX establishment pipeline with 5-fluorouracil, olaparib or combined treatment. **e** The levels of MARVELD1 and PARP1 expression were analyzed by IHC in CRC PDX models. Scale bar, 100 μm. Partial magnification show the localizations of MARVELD1 and PARP1 in the nucleus. **f** PDX tumor sizes are shown after grouping and treatment for 3 weeks. **g**, **h** The relative tumor weight or the inhibition rates after the indicated treatments in low or high MARVELD1 expression PDX models. **i** The relative tumor volumes were measured each 3 days until 21 days in PDX models. *p < 0.05, **p < 0.01. **j** The levels of MARVELD1 and PARP1 expression were analyzed by IHC in PDX models after the indicated treatments. Scale bar, 100 μm. Partial magnification show the localizations of MARVELD1 and PARP1 in the nucleus.

Based on the high MARVELD1 expression presenting worse overall survival and high PARP1 expression also associated with chemo-resistance [17, 33], we further noted that high MARVELD1 expression was associated with poor chemotherapy in COAD, and poor radiotherapy in LGG and GBM (Supplementary Fig. S6a). Thus, we obtained the correlation of MARVELD1 and PARP1 proteins using CRC tissue microarrays (93 pairs of cases). The 93 cases were divided into high expression and low expression group of MARVELD1, the high MARVELD1 expression associated with a worse overall survival, and a positive correlation (*r* value 0.42 and *p* value 0.0032) between MARVELD1 and PARP1 protein expression was shown (Fig. 7a–c).

To elucidate the partnership of MARVELD1 and PARP1 in cancer chemo-resistance, the expression of MARVELD1 and PARP1 protein was assessed in a normal colorectal epithelial cell line (NCM460) and a panel of CRC cell lines (DLD1, HT29, LS174T, T84, HCT15, HCT116, LoVo, RKO) (Supplementary Fig. S6b). And LS174T cells with high expression of MARVELD1 exhibited greater PARP1 stability compared to LoVo cells with low expression of MARVELD1 (Supplementary Fig. S6c). Consistently, stably expressing MARVELD1-Flag also improved PARP1 stability in RKO cells (Supplementary Fig. S6d). The partnership between MARVELD1 and PARP1 was validated in RKO or HCT15 cells (Supplementary Fig. S6e). MARVELD1 also improved the interaction of endogenously expressed PARP1 and NAA50 in these cells (Supplementary Fig. S6f). Furthermore, the MARVELD1 endogenous expression positively correlated with the IC50 of cells treated by oxaliplatin, irinotecan and 5-fluorouracil (Supplementary Fig. S6g). And RKO/MARVELD1-Flag and HCT15/MARVELD1-Flag cells exhibited more resistance to 5-FU compared to control cells (Supplementary Fig. S6h, i). Meanwhile, olaparib improved the sensitivity of cells to 5-FU, including DLD1 cells with MARVELD1 and PARP1 co-expression and the highest IC50 for 5-FU (Supplementary Fig. S6h–j).

After establishing the Patient-derived xenograft (PDX) models of CRC as described in the ‘Materials and Methods’ section, six PDX models with different MARVELD1 levels were selected to carry out experiments (Fig. 7d). The IHC images of CRC tissues proposed for the PDX models showed a positive correlation between MARVELD1 and PARP1 protein levels in high- or low- expression of MARVELD1 group, respectively (Fig. 7e). As shown in Fig. 7f, the tumors of the low MARVELD1 expression group were more sensitive to 5-FU and olaparib combination therapy after 21 days of treatment, while the high MARVELD1 expression group had a poor response to the combination treatment. Because of the heterogeneity and different growth rates, we compared the treatment susceptibility between these models by relative tumor weight, tumor inhibition rate and tumor volume (Fig. 7g–i). These graphs indicated that cases with low MARVELD1 expression were more likely to benefit from therapy with 5-FU or olaparib, especially the combination of the two. The IHC images of PDX model CRC043 and CRC058 after the indicated treatments were shown, representing low or high MARVELD1 expression group, respectively (Fig. 7j). The staining results verified that the expression levels of MARVELD1 and PARP1 are positively related and low MARVELD1 expression refers sensitivity to the combination treatment. The PDX models provide the evidence that the partnership between MARVELD1 and PARP1 could induce therapeutic resistance. And the study offers a clinical guidance of chemotherapeutic drugs combined with PARPi.

## Discussion

Activation of DDR signaling maintains cellular homeostasis and genomic stability in the presence of DNA damage [32–34]. To preserve genome stability and cell survival after both reparable and irreparable lesions, various proteins in DDR system regulate cellular redox to control ROS, promote DNA repair, and prevent cell death [35]. These proteins are also associated with tumorigenesis and resistance to cancer therapy [36]. In this paper, we reveal a clear role of MARVELD1 in regulating DDR network and resisting genotoxic stress-induced DNA damage. MARVELD1 exhibited a typical nuclear localization and interacted with a large number of DDR proteins when cells were stimulated by chemotherapeutic agents (HU). These proteins are also involved in DNA replication licensing factors (3–7 subunits in six factors of MCM), the YWHA family (six members) and regulators of cell death besides oxidation reduction, suggesting that MARVELD1 mediates the DDR process and links with cancer therapy [37–40]. Notably, we observed that MARVELD1 can interact with PARP1, a key sensor of early DDR events [12, 13], and was not dependent on the DNA strand. Given that MARVELD1 bound multiple DDR sensor molecules, we consider that MARVELD1 participates in the regulation of DDR and may be an early event in DDR. Since the protein can bind many specialized “transducers” and “effectors” of the DDR, MARVELD1 is likely a mediator of DDR network, which expands the number and variety of recruited DDR molecules and improves tolerance to DNA damage.

MARVELD1 expression positively correlated with chemotherapy resistance in ovarian cancer [41] and a poor prognosis in gliomas [42], and it was regarded as the genomic instability-derived gene prognostic signature [43]. These studies note the importance of MARVELD1 in maintaining genomic stability and affecting therapeutic efficacy. We observed that the high expression of MARVELD1 reduced cell sensitivity to genotoxic stress, and endogenous MARVELD1 could tolerate IR or UV-induced DNA damage. MARVELD1 knockout mice showed more genome aberration and enhanced sensitivity to IR and KBrO_3_ treatment. Meanwhile, the high expression of MARVELD1 in tumor tissues was closely associated with a poor prognosis in cancer patients. These evidences demonstrate that MARVELD1 is clearly involved in tumorigenesis and resistance to cancer therapy by participating in DDR. DDR has been considered as a candidate anticancer barrier in early tumorigenesis and cancer treatment, known as “a double-edged sword in cancer prevention and cancer therapy” [6, 8]. Therefore, our findings also suggest that MARVELD1 may be a tumor therapeutic intervention protein that acts as a positive guardian of genomic stability through mediating the DDR process.

The recruitment of DDR proteins and molecular choreography at DNA damage sites rely heavily on PTMs, which dynamically regulate protein stability, activity and protein–protein interactions [16]. Under genotoxic stress, PARylation, one of the crucial PTMs associated with DDR, is primarily catalyzed by PARP1 [44]. PAR-dependent events have been implicated in the cellular response to DNA single-strand and double-strand breaks, as well as in maintaining the integrity of perturbed replication forks [45]. However, little is known about PARylation acceptor proteins. Here, we uncover that the translocation of MARVELD1 to the nucleus is dependent on PARylation under genotoxic stress. As a novel PARP1 substrate, MARVELD1 PARylation might be impaired by inhibition of PARP1, and thus it was noted to depend on PARP1. Furthermore, we verified that the major PARylation sites of MARVELD1 by PARP1. When the MARVELD1 3A mutant was expressed, DDR efficacy was greatly compromised. MARVELD1 PARylation offers functional evidence of MARVELD1 involved in the DDR system, and the PARylation sites can serve as a potential target for cancer therapy in clinical trials.

As a critical sensor, PARP1 plays extremely important roles in the DDR network [46]. PARP1 function is strongly dependent on the rapid formation of PAR chains on its substrate and itself to achieve regulation of the target protein stability, localization and new interaction scaffolds of proteins [13]. And PARP1 function requires not only PARylation but also ubiquitination and acetylation [16]. After confirming that PARP1 was capable of MARVELD1 PARylation, we proved that MARVELD1 could regulate the PARP1 stability through decreasing PARP1 ubiquitination levels, and shorten the PARP1 half-life with MARVELD1 deletion. We also certified that NAA50, an N-alpha-acetyltransferase, was a novel acetyltransferase for PARP1 acetylation. MARVELD1 improved the interaction between PARP1 and NAA50 and enhanced PARP1 stability in a NAA50-dependent manner, indicating that MARVELD1 stabilizes PARP1 via the acetylation. We then propose that PARP1 acetylation by NAA50 hinders its ubiquitination sites, thereby inhibiting subsequent proteasomal degradation. Further studies are needed to identify the exact PTM sites and check whether MARVELD1 binding to ubiquitin proteins affects PARP1 degradation under genotoxic stress.

Classically, the DDR exhibits important functions in protecting against cancer by maintaining cellular homeostasis, and defects in the DDR have been exploited in cancer radio/chemotherapy [6, 47]. Recently, proteins in DDR have been identified as promising pathways for targeted cancer therapy [48]. PARPi, the first clinically approved drugs designed to exploit synthetic lethality, have attracted more attention for their potential in cancers [18]. To address the underlying mechanism by which patients with high MARVELD1 expression present poorer overall survival, we investigated the correlation between endogenous MARVELD1 expression and the IC50 of clinical first-line medications (oxaliplatin, irinotecan and 5-fluorouracil) using CRC cell lines. Furthermore, using CRC PDX models, we assessed the tumor suppressive effect of the combination of 5-FU and olaparib to explicate the partnership of MARVELD1 and PARP1. The results suggest that MARVELD1 can be used as a potential biomarker to guide the selection of genotoxic drugs. The efficacy of chemotherapy in patients with low MARVELD1 expression, for example CRC, will be significantly improved when PARPi combination therapy is administered.

**Fig. 8:**
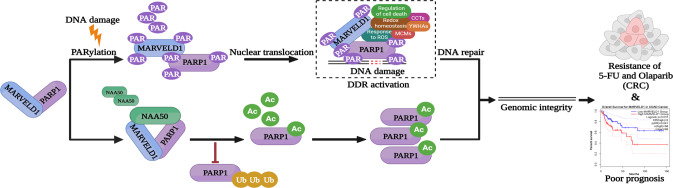
Model for the regulation loop of MARVELD1 interacting with PARP1. PARP1 facilitates the nuclear translocation of MARVELD1 by PARylation to mediate the DDR early events and maintain genome stability. MARVELD1 stabilizes PARP1 by enhancing NAA50-mediated acetylation, thus forming a positive feedback loop. The partnership between MARVELD1 and PARP1 could induce therapeutic resistance.

In conclusion, our work highlights the important role of MARVELD1 in mediating early DDR events to maintain genomic stability, and clarify the partnership of MARVELD1 and PARP1 in promoting therapeutic resistance to genotoxic drugs. Mechanistically, MARVELD1 stabilizes PARP1 by enhancing NAA50-mediated acetylation pathway, and in turn, PARP1 PARylates MARVELD1 which is a novel PARylation acceptor, thus forming a regulation feedback loop (Fig. 8). Our data advance new insights into the clinical guidance of PARPi and therapeutic strategies targeting the DDR in cancer treatment.

## Materials and methods

### Cell culture and transfection

HeLa, HEK293T and SiHa cell lines were purchased from the American Type Culture Collection (ATCC). The cells were cultured in Dulbecco’s modified Eagle’s medium (DMEM, Gibco, USA.) with 10% fetal bovine serum (FBS, Gibco) at 37 °C in a 5% CO_2_ incubator. Wild type mouse embryonic fibroblast (*MARVELD1*^+/+^MEF) and MARVELD1 knockout mouse embryonic fibroblast (*MARVELD1*^−/−^MEF) cells were obtained following procedures described previously [19].

Human colon epithelial cell line NCM460 was purchased from iCell Bioscience. Human CRC cell lines DLD1, HT29, LS174T, T84, HCT15, HCT116, LoVo and RKO were purchased from ATCC. Cell lines NCM460 and DLD1 were cultured in RPMI-1640 medium, HT29 and HCT116 were cultured in McCoy’s 5 A medium, LS174T and RKO were cultured in MEM medium, T84 were cultured in DMEM/F-12 (1:1) medium, HCT15 were cultured in DMEM medium, LoVo were cultured in F12K medium. All media for the human CRC cell lines were purchased from Gibco and supplemented with 10% fetal bovine serum (FBS, Gibco) at 37 °C in a 5% CO_2_ incubator. All cell lines were validated by STR DNA finger printing. Mycoplasma contamination was ruled out by a PCR-based method.

Cell transfections were performed using Lipofectamine 2000 (Invitrogen, USA) according to manufacturer’s instructions.

### Real-time quantitative PCR

Total RNA was extracted using Trizol reagent (Invitrogen, USA) and reversed to cDNA by PrimeScript RT Master Mix (Takara, China). Real time quantitative PCR analyses were performed in triplicate using FastStart Universal SYBR Green Master (Roche, Germany). Fold changes of the target genes were normalized to GAPDH expression by the method of comparative Ct. Primer information was described in Supplementary Table S3.

### Plasmids and siRNAs

MARVELD1-V5, MARVELD1-Flag, and HA-ubiquitin were constructed. PARP1-Flag and NAA50-Flag plasmids were purchased from Vigene Biosciences (Jinan, China). PARP1 truncated mutants were subcloned into pENTER, NAA50 was cloned into pCMV-Myc to generate Myc-tagged recombinant. MARVELD1 and MARVELD1 mutant were cloned into pLVSIN-CMV to generate EGFP tagged recombinants. All construct sequences were verified by DNA sequencing. The detailed information about expression constructs was provided in Supplementary Table S4. Small interfering RNAs (siRNA) targeting PARP1, NAA50, MARVELD1 and corresponding negative control siRNAs (siNC) were purchased from GenePharma Company (Shanghai, China). The sequences of siRNAs were as Supplementary Table S5.

### Antibodies, western blot and co-immunoprecipitation

The detailed information of antibodies included in this study was provided in Supplementary Table S6. Western Blot and co-immunoprecipitation were carried out as described previously [19]. The relative protein level was marked below the band.

### In vivo ubiquitination assays

HA-ubiquitin, PARP1-Flag, and other indicated plasmid were transfected into cells. After transfection 42 h, cells were treated with 10 μM MG132 (Selleckchem, USA) for 6 h, then lysed in RIPA buffer contained 1% SDS (50 mM Tris-HCl, pH 7.5, 150 mM NaCl, 1% NP-40, 1% SDS), and subjected to sonication. After centrifugation (12000 rpm, 15 min, 4 °C), the cell lysates were divided into two parts, IP and input. The IP samples were incubated with anti-DYKDDDDK magnetic agarose (Thermo, USA) at room temperature for 4 h. After washing five times with PBS buffer, samples were boiled in 2× SDS loading buffer. The input samples were boiled in 5× SDS loading buffer. All the samples were resolved in SDS-PAGE for immunoblot analysis.

### Immunofluorescence staining

Cells were seeded on poly-lysine-coated coverslips and subject to indicated treatment. Cells were fixed in 4% paraformaldehyde for 15 min and permeabilized in 1% Triton X-100 solution for 5 min at room temperature. Cells were then blocked with blocking buffer (5% bovine serum albumin in PBS) for 1 h at room temperature and incubated with indicated primary antibody overnight at 4 °C. The coverslips were washed three times with PBS buffer, and the secondary antibody was applied for 0.5 h at 37 °C. Nuclei were stained with DAPI. The coverslips were then mounted onto glass slides using an antifade mounting medium (Beyotime, China). Cells were then examined under a laser scanning confocal microscope (Zeiss LSM 510 Meta). The scale bars as shown in the Figure legends.

The method of fluorescence intensity quantification was described previously with some modifications [49–51]. Image J software was used to perform immunofluorescence quantification as followed: use the lasso tool to draw the nucleus area where the fluorescence intensity is to be measured, and measure its total fluorescence intensity and area. The ratio of total fluorescence intensity to area is the relative nuclear MARVELD1 amount and 50 cells were measured per treatment.

### Immunohistochemical staining

A CRC tissue microarray containing samples from 93 cases paired adjacent noncancerous tissue was purchased from Superchip Biotech (HColA180Su18) (Shanghai, China). The livers and kidneys from the whole-body irradiated mice were embedded and made into 5-μm-thick sections. After deparaffinizing, the sections were heated in citrate buffer (0.01 M), treated with 0.3% H_2_O_2_, blocked with 3% bovine serum albumin solution and incubated with the indicated primary antibody overnight at 4 °C. The sections were incubated in the biotinylated secondary antibody, exposed to diaminobenzidine, and counterstained with Hematoxylin. After serial dehydration, the slides were detected for microscopic.

The immunohistochemistry (IHC) scoring method refers to the immunoreactive score (IRS) system [52]. The intensity of MARVELD1 staining was scored as 0 (no signal), 1 (weak), 2 (moderate), and 3 (marked), and the percentage of stained cells in the whole area was scored as (1) 0–25%; (2) 26–50%; (3) 51–75%; and (4) 76–100%. The scores of each tumor sample were multiplied to give a final score of 0–14, and the samples were determined according to their MARVELD1 expression as low expression, score < 9, and positive expression, score ≥ 9. The staining intensity was graded independently three times in all cases. The scale bars as shown in the Figure legends.

### Detection of PARylated proteins

Detection of PARylated proteins was performed under denaturing conditions as described previously with some modifications [53]. Cells were lysed in RIPA denaturing buffer (50 mM Tris-HCl, pH 8.0, 5 mM EDTA, 150 mM NaCl, 0.5% NP-40, 0.5% deoxycholate, 0.5% SDS) containing 10 μM PARG inhibitor PDD00017273 (Selleckchem, USA), benzonase (Alphabio, China) and protease inhibitor (Bimake, USA). The samples were incubated at 4 °C for 1 h on a rotating platform. Lysates were then immunoprecipitated using anti-DYKDDDDK magnetic agarose (Thermo, USA) at room temperature for 4 h. After washing five times with PBS buffer, samples were boiled in 2× SDS loading buffer and resolved in SDS-PAGE. Immunoblotting analysis was carried out with an anti-PAR monoclonal antibody (R&D systems, USA).

### LC-MS/MS and Proteomic analysis

To analyze MARVELD1 interacting proteins, HeLa cells were transfected with MARVELD1-Flag or pcDNA3.1 (PC), then treated with 8 mM Hydroxyurea (MCE, USA) or DMSO for 24 h, and subjected to IP assays with anti-DYKDDDDK magnetic agarose (Thermo, USA). After washing five times with PBS buffer, samples were boiled in 2× SDS loading buffer, resolved in SDS-PAGE, visualized by Coomassie Blue staining, and subjected to liquid chromatography-tandem mass spectrometry (LC-MS/MS) analysis (PTM Bio, China, project number: FA203GI). Simultaneously, we stained the gel using Silver Stain Kit (CoWin Biosciences, China) after SDS-PAGE finished. The staining assays were carried out as described previously [54].

Compared with the control group, peptides with a ratio of more than 2 were more likely to interact with MARVELD1 in the mass spectrometry data. Used the R package “cluster Profiler” to perform GO function annotation and KEGG enrichment analysis with BH-corrected *p* < 0.05 to select the main functional and biological pathways of genes were enriched. Protein-protein interactions (PPIs) were obtained from String database (https://cn.string-db.org/) and Human Genome Wide Network Compendium database (https://www.ndexbio.org/). All the statistical analyses were performed using R Statistical Software (V-4.0), and biological networks were visualized by “Cytoscape”. Squares or rectangles display functions, the red dots represent HU-specific proteins, and the orange dots represent the proteins whose ratio of the HU group to the control group is greater than 2.

### Cell viability and colony-formation assay

Cells were seeded in 96-well plates (1000 cells per well) in sextuplicate with indicated treatment. Cell viability was determined by Cell Counting Kit-8 (CCK-8) (Bimake, USA) according to the manufacturer’s instructions. Data were shown as the mean with SD. For colony-formation assay, cells were seeded in 6-well plates (500 cells per well) in triplicate and cultured under indicated treatment for 15 days. Colonies were stained with 1% Crystal Violet, taken photographs, and counted. Colony numbers were presented as the mean with SD of three independent experiments.

### Cell cycle synchronization and analysis

HeLa/MARVELD1 and HeLa/PC cells were synchronized at the G1/S boundary with TDR, a drug that synchronizes the cell cycle. HeLa cells were treated with TDR for 16 h, released for 10 h and following with TDR treatment for the second time. After removing the TDR, HeLa cells were cultured with HU for 4 h.

Cells with different treatment were trypsinized, washed once with PBS and fixed with 70% ethanol overnight at −20 °C. After fixation, cells were washed once with PBS following staining with PI/RNAse staining solution (Tianjin Sungene Biotech, China) at room temperature for 30 min. The stained cells were re-suspended in PBS. Flow cytometry (FCM) analysis was performed with a flow cytometer (BD Biosciences, USA).

### Neutral comet assay

After treatment with or without Hydroxyurea (MCE, USA) for indicated time, cells were collected for measuring the DNA damage level by comet assay. The assays were carried out as described previously [54]. Representative images and quantified tail moments were shown for each group, *n* > 60 cells from each sample. The graphs showed the mean with SD.

### Nuclear and cytoplasmic protein extraction and micrococcal nuclease sensitivity assays

HeLa cells were treated with the indicated conditions and washed twice with PBS. Cells were suspended in buffer A (10 mM HEPES, 10 mM KCl, 1.5 mM MgCl_2_, 0.34 M sucrose, 10% glycerol, 1 mM DTT, 1% protease inhibitors, 0.1% Triton X-100) for 5 min on ice. After centrifugation at 1300 rpm for 5 min at 4 °C, the supernatant was the cytoplasmic protein and precipitation was the nucleus protein. The isolated nuclei were washed twice using buffer A, and then re-suspended in buffer B (3 mM EDTA, 0.2 mM EGTA, 1 mM DTT, 1% protease inhibitors) for 10 min on ice before sonication for 20 s and centrifugation at 1300 rpm for 5 min. The cytoplasmic or nucleus proteins were boiled in 5× SDS loading buffer and resolved in SDS-PAGE for immunoblot analysis.

Nuclei were suspended in buffer B for 15 min on ice and centrifuged at 1700 rpm for 5 min at 4 °C. The precipitation was the insoluble chromatin.

The insoluble chromatin fraction was resuspended in buffer A containing 20 U/μl MNase (New England Biolabs, China). After incubation at 37 °C for 20 min, the nuclease reaction was stopped by the addition of 5 mM EGTA and then subjected to analysis using 1% native agarose gel electrophoresis.

### Generation of MARVELD1-null mice and whole-body radiation

The whole body MARVELD1-KO mice were generated from C57BL/6 J strains as described previously [55]. Five to eight-week-old *MARVELD1*^+/+^, *MARVELD1*^+/−^, *MARVELD1*^−/−^ littermates were radiated to 5 Gy X-ray. Mice were observed daily after radiation for survival. Observation of the mice was continued for 30 days. The number of animals per group was based on the availability of mice of the different genotypes. In this study, 27 *MARVELD1*^+/+^ mice, 23 *MARVELD1*^+/−^ mice, and 12 *MARVELD1*^−/−^ mice were included. Investigators undertaking the animal monitoring were blinded to the genotype of the mice.

### Analysis of metaphase chromosomes

MEF cells were seeded to approximate 50% confluence. Cells were incubated in 5 μM Nocodazole (MCE, USA) for 12 h. We tapped the culture dish to make the M-phase cells detached. The cells were collected with centrifugation and then incubated in hypotonic solution (0.075 M KCl) at 37 °C for 10 min. Next, cells were fixed in fixative (methanol: acetic acid = 3:1, v/v) at room temperature for 20 min and spread onto pre-cold glass slides and dried. The slides were stained with DAPI and 100 metaphases for each sample were analyzed. Quantification of aberrations was the mean of three independent experiments, and 100 metaphase cells per experiment were counted. Two-tailed Student’s *t* test.

### KBrO_3_ treatment

1500 ppM KBrO_3_ (Sigma-Aldrich, USA) was given to 8 months old *MARVELD1*^+/+^ mice, *MARVELD1*^+/−^ mice, and *MARVELD1*^−/−^ mice in their drinking water for 5 weeks. After 5 weeks, all the mice were sacrificed. The collected blood serum, part of the livers and brains frozen quickly in liquid nitrogen for ELISA assays. The remaining livers and brains were fixed in 4% paraformaldehyde solution for tissue immunofluorescence analysis. In this study, 5 *MARVELD1*^+/+^ mice, 5 *MARVELD1*^+/−^ mice, and 5 *MARVELD1*^−/−^ mice were included. Investigators undertaking the animal monitoring were blinded to the genotype of the mice.

### ELISA

The ELISA assays were performed using Mouse 8-OHdG ELISA Kit (Jingkang Bioengineering, China) according to manufacturer’s instructions. Data were presented as the mean with SD.

### Patient-derived xenograft (PDX) model

PDX model treatment assays refer to our previous research [56]. In brief, tumor fragments were cut into pieces of 2–3 mm in diameter and inoculated subcutaneously into the right flank of the six-week-old female NOD-SCID mice (Vital River Laboratories, Beijing, China). Tumor volumes were recorded every three days according to the formula: (width)^2^ ×height/2. When tumors reached 100–150 mm^3^, the mice (12/group) were randomized into four groups: (i) vehicle (normal saline 100 μl, day 1–5/week by i.p. injection), (ii) 5-Fluorouracil (25 mg/kg 5-Fluorouracil, day 2, 5/week by i.p. injection), (iii) olaparib (50 mg/kg olaparib, day 1–5/week by i.p. injection) and (iv) combination group (25 mg/kg 5-Fluorouracil, day 2, 5/week and 50 mg/kg olaparib, day 1–5/week by i.p. injection). After the administration of the vehicle, 5-Fu and olaparib for 3 weeks, we stopped the treatments and analyzed the tumors size and weight.

Investigators undertaking the animal monitoring were blinded to the genotype of the mice. All animal studies were conducted in accordance with the National Institute of Health guidelines for the Care and Use of Laboratory Animals and approved by the Ethics Committee of Harbin Medical University Cancer Hospital. The graph showed the mean with SD.

### Statistical analysis

The results are presented as the mean ± standard of measurement at least three independent experiments. Statistical significance was determined using a two-tailed Student’s *t* test. And *p* < 0.05 was considered statistically significant (*p < 0.05; **p < 0.01; ***p < 0.001). For each figure, statistical tests are justified as appropriate. Analyses and graphical presentation were performed using the GraphPad Prism 8.0 software.

### Reporting summary

Further information on research design is available in the Nature Research Reporting Summary linked to this article.

## Supplementary information

Informed Patient Consent

Supplementary Figure S1

Supplementary Figure S2

Supplementary Figure S3

Supplementary Figure S4

Supplementary Figure S5

Supplementary Figure S6

Supplementary Figure Legends

Supplementary Table S1-6

Reporting Summary

## Data Availability

Data that support the findings of this study are available from the corresponding author on reasonable request. The mass spectrometry proteomics data have been deposited to the ProteomeXchange Consortium via the PRIDE partner repository with the dataset identifier PXD034445.
