# Supplementary material for: The regulation loop of MARVELD1 interacting with PARP1 in DNA damage response maintains genome stability and promotes therapy resistance of cancer cells
Source: Cell Death Differ. 2023 Feb 7;30(4):922–37. doi: 10.1038/s41418-023-01118-z (PMC10070477; doi:10.1038/s41418-023-01118-z)
Supplement: Supplementary file 1 — Informed Patient Consent [file 41418_2023_1118_MOESM1_ESM.pdf]

哈尔滨医科大学附属肿瘤医院

（黑龙江省肿瘤医院）

病史数据/生物标本二次利用知情同意书

姓名：范丽梅　　性别：女　　年龄：48岁　　科别：内五　　住院号：853143

哈尔滨医科大学附属肿瘤医院收集和储存的病史数据及生物标本，主要用于肿瘤疾病预防和诊治等方面的科学研究项目及调查。

您同意留取标本完全是出于自愿，并且可以随时终止配合此项工作而无须提出任何理由，您的任何医疗待遇与权益不会因此而受到影响。

在帮助您了解以上情况并征得您本人（法定监护人）同意后，在不影响病理诊断、临床检验和治疗的前提下，我们将留取以下您的生物标本，包括：活检或手术切除后的组织、病理/检验/手术后废弃的血液标本、病理/检验/手术后废弃的尿液标本、 病史数据、其他：

所留取的标本及您的相关病史数据信息，将由专业人员进行科学规范的管理。在标本收集、保管和使用过程中，您的个人资料均属保密。可以识别您身份的信息将不会透露给研究小组、政府管理部门或伦理委员会以外的人员，除非获得您的许可。这项研究结果发表时，将不会披露您个人的任何资料。

您既不会从本研究中获得某种形式的经济利益，也不会让您承担各种实验费用。所有资源仅用于科学研究，不做其它目的。

同意声明：

我已阅读上述有关生物标本二次利用的介绍，同时也了解到我有权不参加或在参加过程中可随时退出，都不会因此对治疗造成任何影响。我自愿参加并同意提供以上标本用于科学研究。

患者签名：范丽梅　　2021年04月08日

联系电话：

当患者知情同意能力欠缺或不足时（如18岁以下儿童、精神障碍患者等）增加以下方式：

法定监护人签名：　　与患者关系：　　2021年04月08日

医生签名：赵红丽　　2021年04月08日

哈尔滨医科大学附属肿瘤医院

（黑龙江省肿瘤医院）

病史数据/生物标本二次利用知情同意书

姓名：李淑琴　　性别：女　　年龄：68岁　　科别：结直肠外科二病房　　住院号：852004

哈尔滨医科大学附属肿瘤医院收集和储存的病史数据及生物标本，主要用于肿瘤疾病预防和诊治等方面的科学研究项目及调查。

您同意留取标本完全是出于自愿，并且可以随时终止配合此项工作而无须提出任何理由，您的任何医疗待遇与权益不会因此而受到影响。

在帮助您了解以上情况并征得您本人（法定监护人）同意后，在不影响病理诊断、临床检验和治疗的前提下，我们将留取以下您的生物标本，包括：活检或手术切除后的组织、病理/检验/手术后废弃的血液标本、病理/检验/手术后废弃的尿液标本、 病史数据、其他：

所留取的标本及您的相关病史数据信息，将由专业人员进行科学规范的管理。在标本收集、保管和使用过程中，您的个人资料均属保密。可以识别您身份的信息将不会透露给研究小组、政府管理部门或伦理委员会以外的人员，除非获得您的许可。这项研究结果发表时，将不会披露您个人的任何资料。

您既不会从本研究中获得某种形式的经济利益，也不会让您承担各种实验费用。所有资源仅用于科学研究，不做其它目的。

同意声明：

我已阅读上述有关生物标本二次利用的介绍，同时也了解到我有权不参加或在参加过程中可随时退出，都不会因此对治疗造成任何影响。我自愿参加并同意提供以上标本用于科学研究。

患者签名：李淑琴　　　　2021年04月14日

联系电话：

当患者知情同意能力欠缺或不足时（如18岁以下儿童、精神障碍患者等）增加以下方式：

法定监护人签名：　　　与患者关系：　　　年　月　日

2021年04月14日

医生签名：吴迪

哈尔滨医科大学附属肿瘤医院

（黑龙江省肿瘤医院）

病史数据/生物标本二次利用知情同意书

姓名：邹洪伟　　性别：男　　年龄：48岁　　科别：结直肠外科二病房　　住院号：833360

哈尔滨医科大学附属肿瘤医院收集和储存的病史数据及生物标本，主要用于肿瘤疾病预防和诊治等方面的科学研究项目及调查。

您同意留取标本完全是出于自愿，并且可以随时终止配合此项工作而无须提出任何理由，您的任何医疗待遇与权益不会因此而受到影响。

在帮助您了解以上情况并征得您本人（法定监护人）同意后，在不影响病理诊断、临床检验和治疗的前提下，我们将留取以下您的生物标本，包括：活检或手术切除后的组织、病理/检验/手术后废弃的血液标本、病理/检验/手术后废弃的尿液标本、 病史数据、其他：

所留取的标本及您的相关病史数据信息，将由专业人员进行科学规范的管理。在标本收集、保管和使用过程中，您的个人资料均属保密。可以识别您身份的信息将不会透露给研究小组、政府管理部门或伦理委员会以外的人员，除非获得您的许可。这项研究结果发表时，将不会披露您个人的任何资料。

您既不会从本研究中获得某种形式的经济利益，也不会让您承担各种实验费用。所有资源仅用于科学研究，不做其它目的。

同意声明：

我已阅读上述有关生物标本二次利用的介绍，同时也了解到我有权不参加或在参加过程中可随时退出，都不会因此对治疗造成任何影响。我自愿参加并同意提供以上标本用于科学研究。

患者签名：邹洪伟　　2021年05月10日

联系电话：

当患者知情同意能力欠缺或不足时（如18岁以下儿童、精神障碍患者等）增加以下方式：

法定监护人签名：　　与患者关系：　　2021年05月10日

2021年05月10日

医生签名：吴迪

哈尔滨医科大学附属肿瘤医院

（黑龙江省肿瘤医院）

病史数据/生物标本二次利用知情同意书

姓名：张立荣　　性别：女　　年龄：66岁　　科别：结直肠外科二病房　　住院号：855117

哈尔滨医科大学附属肿瘤医院收集和储存的病史数据及生物标本，主要用于肿瘤疾病预防和诊治等方面的科学研究项目及调查。

您同意留取标本完全是出于自愿，并且可以随时终止配合此项工作而无须提出任何理由，您的任何医疗待遇与权益不会因此而受到影响。

在帮助您了解以上情况并征得您本人（法定监护人）同意后，在不影响病理诊断、临床检验和治疗的前提下，我们将留取以下您的生物标本，包括：活检或手术切除后的组织、病理/检验/手术后废弃的血液标本、病理/检验/手术后废弃的尿液标本、 病史数据、其他：

所留取的标本及您的相关病史数据信息，将由专业人员进行科学规范的管理。在标本收集、保管和使用过程中，您的个人资料均属保密。可以识别您身份的信息将不会透露给研究小组、政府管理部门或伦理委员会以外的人员，除非获得您的许可。这项研究结果发表时，将不会披露您个人的任何资料。

您既不会从本研究中获得某种形式的经济利益，也不会让您承担各种实验费用。所有资源仅用于科学研究，不做其它目的。

同意声明：

我已阅读上述有关生物标本二次利用的介绍，同时也了解到我有权不参加或在参加过程中可随时退出，都不会因此对治疗造成任何影响。我自愿参加并同意提供以上标本用于科学研究。

患者签名：张立荣　　　　2021年04月27日

联系电话：

当患者知情同意能力欠缺或不足时（如18岁以下儿童、精神障碍患者等）增加以下方式：

法定监护人签名：　　　与患者关系：　　　2021年04月27日

医生签名：吴迪　　　　2021年04月27日

哈尔滨医科大学附属肿瘤医院

（黑龙江省肿瘤医院）

病史数据/生物标本二次利用知情同意书

姓名：李伟　　性别：男　　年龄：52岁　　科别：结直肠外科二病房　　住院号：866588

哈尔滨医科大学附属肿瘤医院收集和储存的病史数据及生物标本，主要用于肿瘤疾病预防和诊治等方面的科学研究项目及调查。

您同意留取标本完全是出于自愿，并且可以随时终止配合此项工作而无须提出任何理由，您的任何医疗待遇与权益不会因此而受到影响。

在帮助您了解以上情况并征得您本人（法定监护人）同意后，在不影响病理诊断、临床检验和治疗的前提下，我们将留取以下您的生物标本，包括：活检或手术切除后的组织、病理/检验/手术后废弃的血液标本、病理/检验/手术后废弃的尿液标本、 病史数据、其他：

所留取的标本及您的相关病史数据信息，将由专业人员进行科学规范的管理。在标本收集、保管和使用过程中，您的个人资料均属保密。可以识别您身份的信息将不会透露给研究小组、政府管理部门或伦理委员会以外的人员，除非获得您的许可。这项研究结果发表时，将不会披露您个人的任何资料。

您既不会从本研究中获得某种形式的经济利益，也不会让您承担各种实验费用。所有资源仅用于科学研究，不做其它目的。

同意声明：

我已阅读上述有关生物标本二次利用的介绍，同时也了解到我有权不参加或在参加过程中可随时退出，都不会因此对治疗造成任何影响。我自愿参加并同意提供以上标本用于科学研究。

患者签名： 李伟　　2021年07月02日

联系电话：

当患者知情同意能力欠缺或不足时（如18岁以下儿童、精神障碍患者等）增加以下方式：

法定监护人签名：　　与患者关系：　　2021年07月02日

医生签名： 吴迪　　2021年07月02日

哈尔滨医科大学附属肿瘤医院

（黑龙江省肿瘤医院）

病史数据/生物标本二次利用知情同意书

姓名：孙永绪　性别：男　年龄：50岁　科别：结直肠外科二病房　住院号：866691

哈尔滨医科大学附属肿瘤医院收集和储存的病史数据及生物标本，主要用于肿瘤疾病预防和诊治等方面的科学研究项目及调查。

您同意留取标本完全是出于自愿，并且可以随时终止配合此项工作而无须提出任何理由，您的任何医疗待遇与权益不会因此而受到影响。

在帮助您了解以上情况并征得您本人（法定监护人）同意后，在不影响病理诊断、临床检验和治疗的前提下，我们将留取以下您的生物标本，包括：活检或手术切除后的组织、病理/检验/手术后废弃的血液标本、病理/检验/手术后废弃的尿液标本、 病史数据、其他：

所留取的标本及您的相关病史数据信息，将由专业人员进行科学规范的管理。在标本收集、保管和使用过程中，您的个人资料均属保密。可以识别您身份的信息将不会透露给研究小组、政府管理部门或伦理委员会以外的人员，除非获得您的许可。这项研究结果发表时，将不会披露您个人的任何资料。

您既不会从本研究中获得某种形式的经济利益，也不会让您承担各种实验费用。所有资源仅用于科学研究，不做其它目的。

同意声明：

我已阅读上述有关生物标本二次利用的介绍，同时也了解到我有权不参加或在参加过程中可随时退出，都不会因此对治疗造成任何影响。我自愿参加并同意提供以上标本用于科学研究。

患者签名：孙永绪　　2021年07月02日

联系电话：

当患者知情同意能力欠缺或不足时（如18岁以下儿童、精神障碍患者等）增加以下方式：

法定监护人签名：　与患者关系：　2021年07月02日

医生签名：吴迪　　2021年07月02日
