# Supplementary figures and images for: The regulation loop of MARVELD1 interacting with PARP1 in DNA damage response maintains genome stability and promotes therapy resistance of cancer cells
Source: Cell Death Differ. 2023 Feb 7;30(4):922–37. doi: 10.1038/s41418-023-01118-z (PMC10070477; doi:10.1038/s41418-023-01118-z)

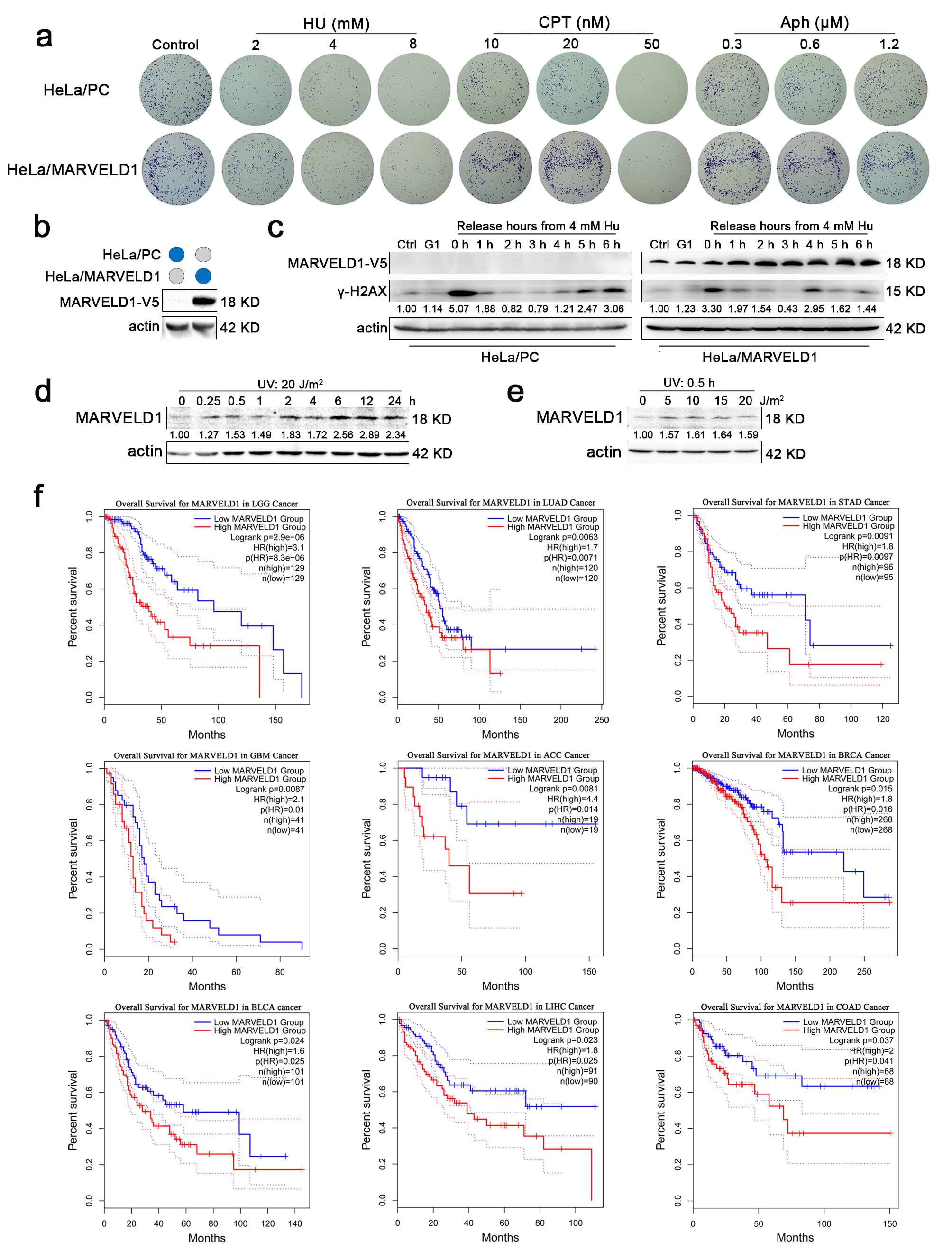

Supplement: Supplementary file 2 — Supplementary Figure S1 [file 41418_2023_1118_MOESM2_ESM.tif]

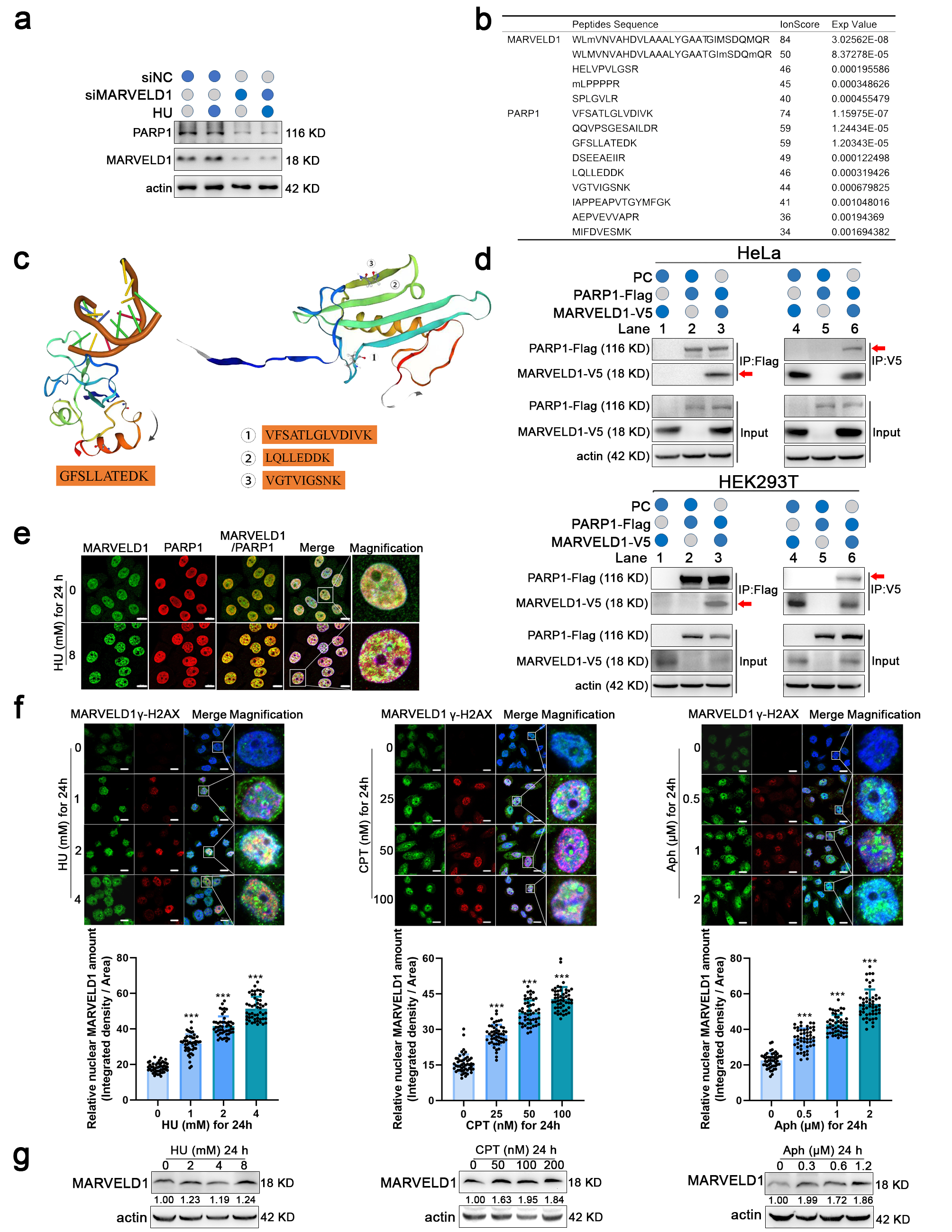

Supplement: Supplementary file 3 — Supplementary Figure S2 [file 41418_2023_1118_MOESM3_ESM.tif]

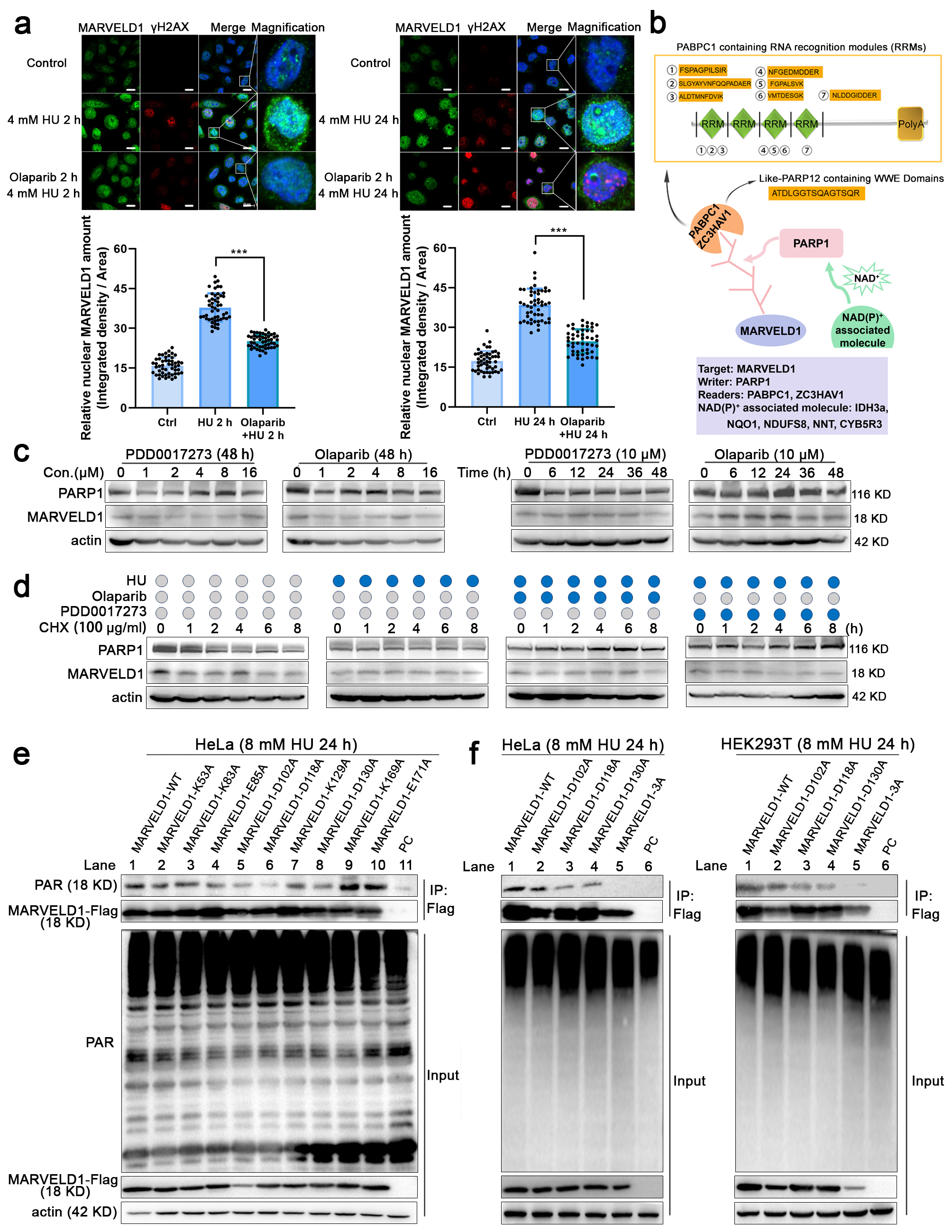

Supplement: Supplementary file 4 — Supplementary Figure S3 [file 41418_2023_1118_MOESM4_ESM.tif]

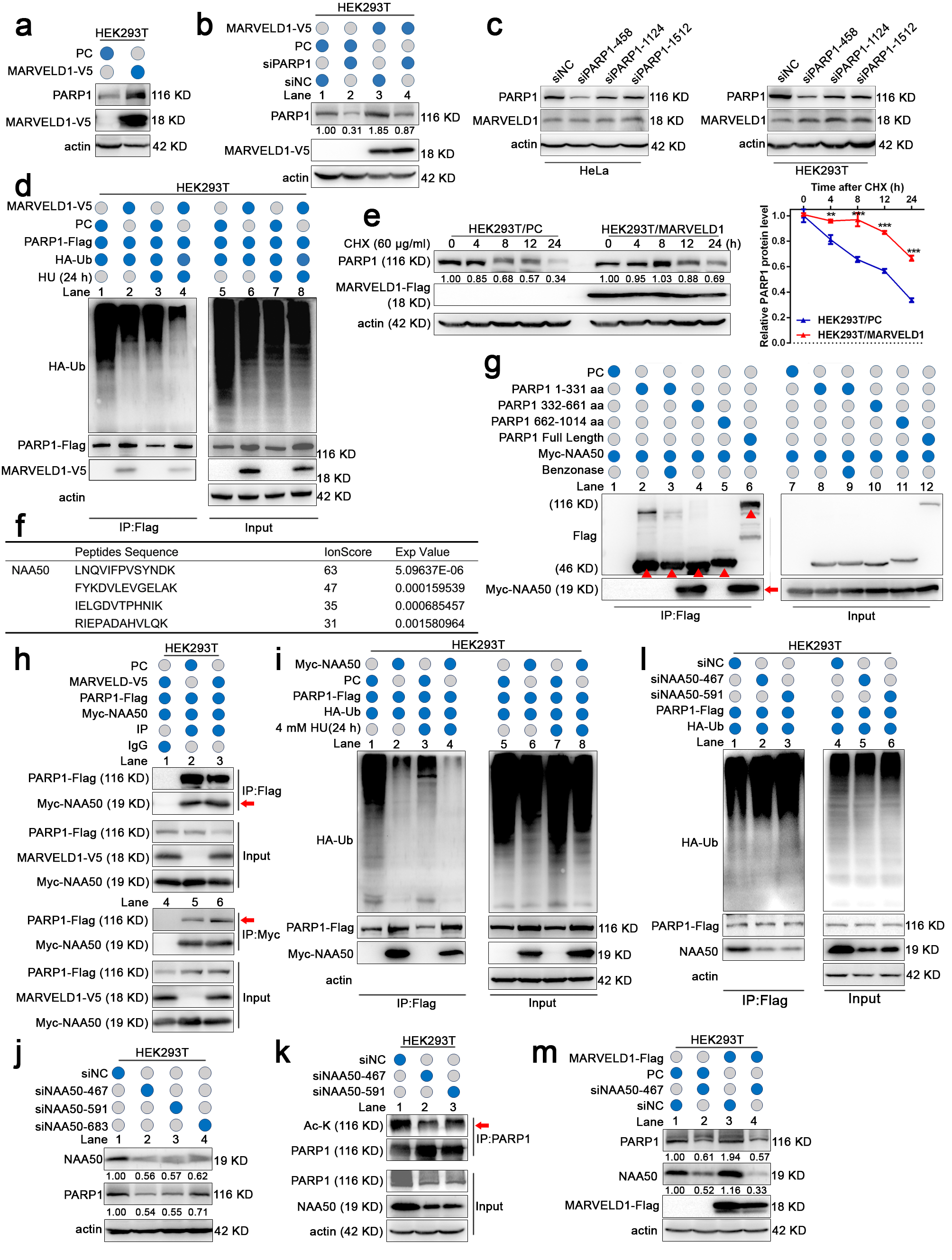

Supplement: Supplementary file 5 — Supplementary Figure S4 [file 41418_2023_1118_MOESM5_ESM.tif]

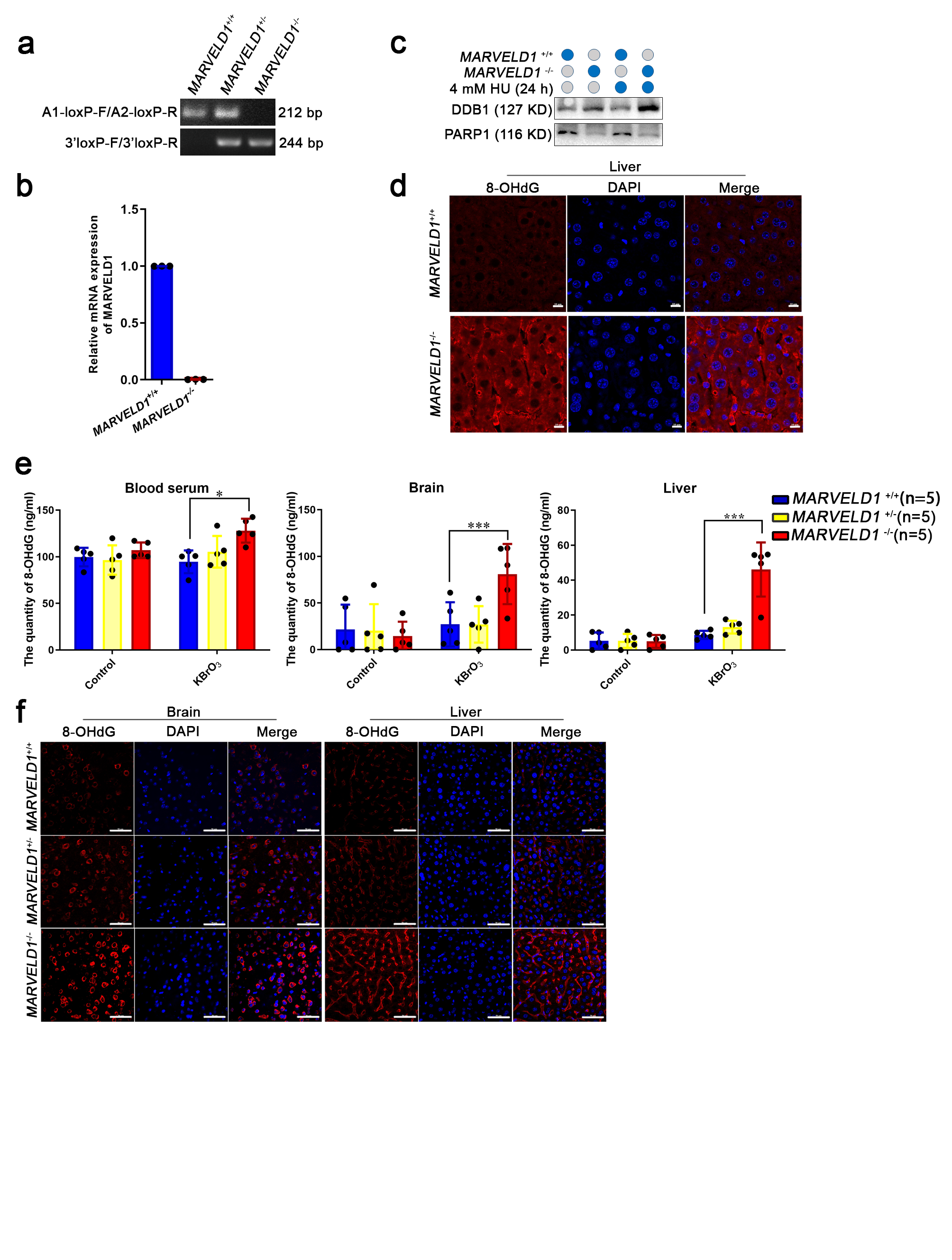

Supplement: Supplementary file 6 — Supplementary Figure S5 [file 41418_2023_1118_MOESM6_ESM.tif]

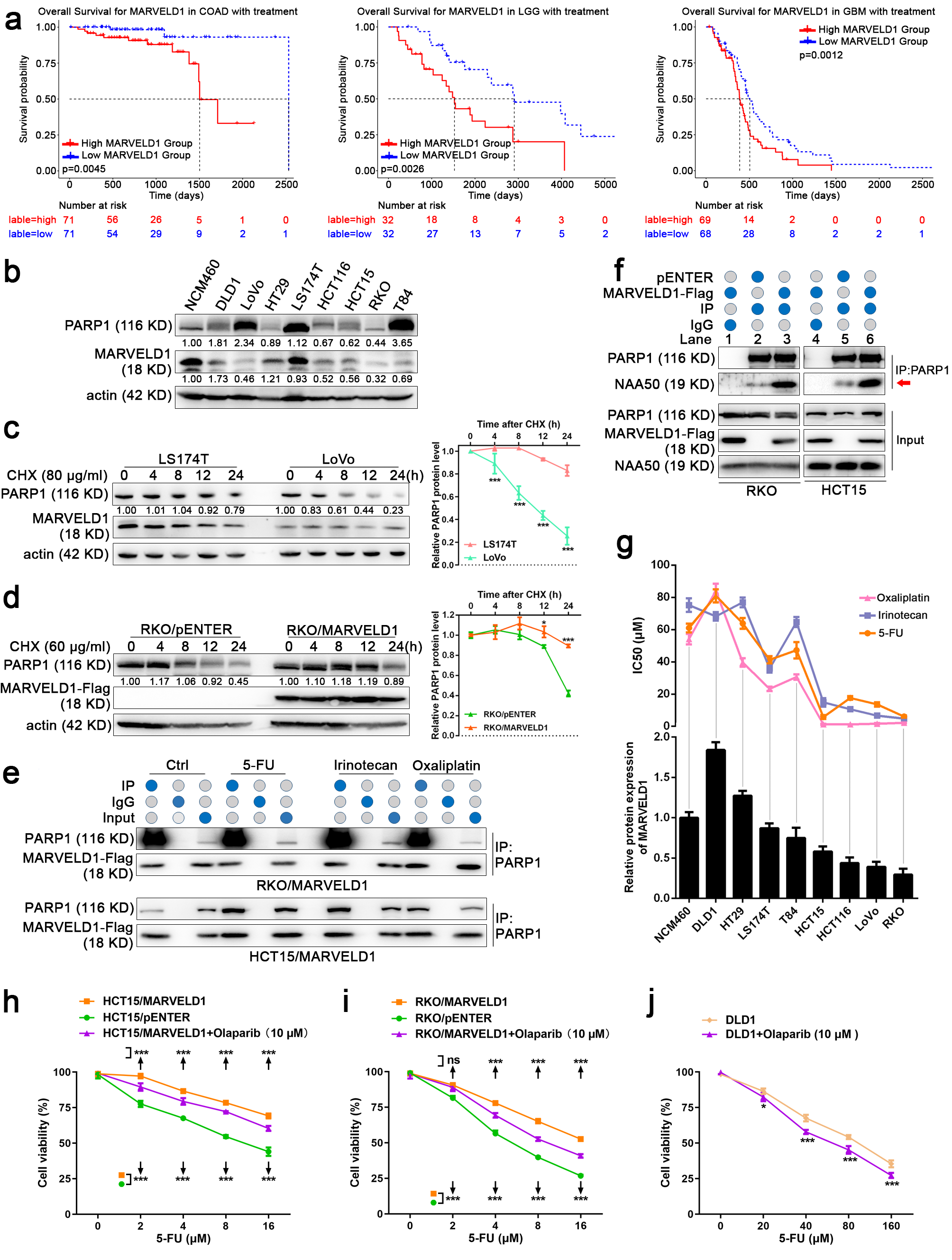

Supplement: Supplementary file 7 — Supplementary Figure S6 [file 41418_2023_1118_MOESM7_ESM.tif]
