# Supplementary material for: The regulation loop of MARVELD1 interacting with PARP1 in DNA damage response maintains genome stability and promotes therapy resistance of cancer cells
Source: Cell Death Differ. 2023 Feb 7;30(4):922–37. doi: 10.1038/s41418-023-01118-z (PMC10070477; doi:10.1038/s41418-023-01118-z)
Supplement: Supplementary file 8 — Supplementary Figure Legends [file 41418_2023_1118_MOESM8_ESM.docx]

**Supplementary Figure legends**

**Supplementary Figure 1**

**a,** Colony-formation assays were performed by HeLa/PC and HeLa/MARVELD1 cells which were seeded at low confluence and grown in the indicated treatment for 14 days. **b,** The HeLa cells stably expressing MARVELD1-V5 were identified by WB. **c,** DNA damage marker (γH2AX) was detected in HeLa/PC or HeLa/MARVELD1 cells. HeLa/PC cells showed higher γH2AX protein levels than HeLa/MARVELD1 cells at 0 h after release. The higher expression of γH2AX in the HeLa/PC group lasted over 6 h, while in the HeLa/MARVELD1 group, γH2AX began to decrease 4 h after release, consistent with the cell cycle progression. **d,** After 20 J/m^2^ UV radiation, endogenous MARVELD1 protein levels in HeLa cells were assessed by WB in 24 h. **e,** After treatment with the indicated dose of UV radiation for 0.5 h, endogenous MARVELD1 protein levels in HeLa cells were assessed by WB. **f,** The OS of patients with LGG, LUAD, STAD, GBM, ACC, BRCA, BLCA, LIHC and COAD were analyzed using Kaplan–Meier analysis based on MARVELD1 expression in the GEPIA dataset. **p*<0.05, ***p*<0.01, ****p*<0.001.

**Supplementary Figure 2**

**a,** The MARVELD1 knockdown efficiency was analyzed by WB in HeLa cells. **b,** The identified interacting peptides for MARVED1 and PARP1 are shown. **c,** Prediction of protein structures with the SWISS-MODEL online tool for PARP1 peptides interacting with MARVELD1, including the DNA binding motif (GFSLLATEDK) and reverse parallel sequences (VFSATLGLVDIVK; LQLLEDDK; VGTVIGSNK). **d,** The interaction of MARVELD1 and PARP1 was analyzed by IP with IgG or the indicated antibody in HeLa and HEK293T cells, followed by WB. **e,** IF staining of HeLa cells with MARVELD1 or PARP1 antibodies after the indicated treatments. Scale bar: 10 μm. **f,** MARVELD1 clustered in nucleus was analyzed by IF staining when SiHa cells were treated by different dose of HU, CPT or Aph. Green staining indicates MARVELD1, red staining indicates γ-H2AX, blue staining indicates DAPI. The relative nuclear MARVELD1 amount (integrated density/area) per cell was quantified by ImageJ and 50 cells were measured per treatment. Scale bar: 10 μm.****p*<0.001. **g,** The endogenous MARVELD1 protein level was assessed in HeLa cells treated with HU, CPT or Aph for the indicated dose and time.

**Supplementary Figure 3**

**a,** The MARVELD1 subcellular location was detected by IF staining in SiHa cells treated with olaparib or HU. The relative nuclear MARVELD1 amount (integrated density/area) per cell was quantified by ImageJ and 50 cells were measured per treatment. Scale bar: 10 μm. The data are shown as the mean with SD. ****p*<0.001. **b,** Schematic diagram of PARylation-associated molecules among MARVELD1-interacting proteins. The PABPC1 peptides interacting with MARVELD1 was located in the RNA recognition modules (RRMs). **c,** MARVELD1 and PARP1 protein levels were analyzed in HeLa cells treated with PDD0017273 or olaparib by WB. **d,** The half-life of MARVELD1 in HeLa cells was detected when cells were treated with HU, olaparib or PDD0017273. **e-f,** MARVELD1 PARylation sites were identified in HeLa and HEK293T cells transfected with the indicated expression vectors. After transfection, lysates were subjected to IP and WB analyses with the PAR antibody.

**Supplementary Figure 4**

**a,** PARP1 protein level in HEK293T/MARVELD1 cells. **b,** PARP1 protein level was analyzed by WB when MARVELD1-V5 and PARP1 siRNA was co-transfected in HEK293T cells after 48 h. **c,** PARP1 and MARVELD1 protein levels were tested in HeLa and HEK293T cells transfected with the PARP1 siRNA. **d,** The Ubiqutin-PARP1 was analyzed in HEK293T cells. After cells treated with or without 4mM HU for 24 h, MG-132 was added for 6 h, and lysates were subjected to IP and WB analysis. **e,** The half-life of PARP1 was detected by WB in HEK293T cells treated with CHX for the indicated time points. Relative PARP1 protein levels (PARP1/actin) were quantified and plotted in the right panels. ***p*<0.01, ****p*<0.001. **f,** The identified peptides of NAA50 responsible for interaction with MARVELD1 protein are shown. **g,** Truncated PARP1-flag and Myc-NAA50 were co-transfected into HEK293T cells. Lysates were immunoprecipitated with Flag antibody. **h,** The effect of MARVELD1 on the interaction of PARP1 and NAA50 was tested by IP at 48 h after HEK293T cells were transfected. **i,** The Ubiqutin-PARP1 level was checked in HEK293T/NAA50-Flag cells treated with or without 4 mM HU for 24 h. After HU treatment, cells were treated with MG-132 for 6 h, and lysates were subjected to IP and WB analysis. **j-k,** PARP1 protein and acetylation level was detected in HEK293T/siNAA50 cells after 48h transfection. **l,** The Ubiqutin-PARP1 level was verified in HEK293T/siNAA50 cells. At 48 h transfection, HEK293T cells were treated with MG-132 for 6 h, and lysates were subjected to IP and WB analysis. **m,** PARP1 protein level was analyzed in HEK293T/MARVELD1 cells transfected with the NAA50 siRNA. WB was performed after 48 h transfection.

**Supplementary Figure 5**

**a,** MARVELD1 KO mice were verified by genotyping. Mouse genomic DNA was extracted from the tails of newborn mice. **b,** The quantitative results of the mRNA level of MARVELFD1 in *MARVELD1*^+/+^ or *MARVELD1*^-/-^MEFs are presented as the mean with SD from three independent experiments. **c,** DDB1 and PARP1 protein levels were detected in *MARVELD1*^+/+^ or *MARVELD1*^-/-^ MEFs by WB. **d,** Representative 8-OHdG IF staining of the indicated tissues from 5 Gy X-ray-irradiated *MARVELD1*^+/+^ or *MARVELD1*^-/-^ mice is shown. Scale bar: 10 μm. **e,** *MARVELD1*^+/+^ (n=5), *MARVELD1*^+/-^ (n=5), and *MARVELD1*^-/-^ (n=5) mice were treated with 1,500 ppm KBro_3_ in drinking water for five weeks. ELISAs were performed to determine the levels of 8-OHdG in blood serum, brain, and liver. Data are presented as the mean with SD. **p*<0.05, ****p*<0.001. **f,** Representative 8-OHdG IF staining of the indicated tissues from KBrO_3_-treated *MARVELD1*^+/+^ or *MARVELD1*^-/-^ mice is shown. Scale bar: 50 μm.

**Supplementary Figure 6**

**a,** The OS of COAD patients with chemotherapy, LGG or GBM patients with radiotherapy were analyzed using Kaplan–Meier analysis based on MARVELD1 expression through TCGA dataset. **b,** Endogenous PARP1 and MARVELD1 protein levels in the human colon epithelial cell line NCM460 and 8 CRC cell lines were assessed by WB. **c-d,** The half-life of PARP1 was detected by WB in LS174T, LoVo or stably expressing MARVELD1-Flag RKO cells treated with CHX for the indicated time points. Relative PARP1 protein levels (PARP1/actin) were quantified and plotted in the lower panels. *p<0.05, ***p<0.001. **e,** The interactions of PARP1 and MARVELD1 were detected in RKO or HCT15 cells stably expressing MARVELD1-Flag under the indicated treatments. **f,** The effect of MARVELD1 on the interaction of PARP1 and NAA50 was tested by IP in RKO or HCT15 cells stably expressing MARVELD1-Flag. **g,** IC50 analysis of oxaliplatin, irinotecan and 5-FU and quantitative results of MARVELD1 protein levels in the human colon epithelial cell line NCM460 and 8 CRC cell lines. **h-i,** Cell viability assays of RKO or HCT15 cells stably expressing MARVELD1-Flag in response to increasing doses of 5-FU combined with olaparib for 48 h were performed. Data are presented as the mean with SD of three independent experiments. ****p*<0.001. **j,** Cell viability assays of DLD1 cells in response to increasing doses of 5-FU combined with olaparib for 48 h were performed. Data are presented as the mean with SD of three independent experiments. **p*<0.05, ****p*<0.001.
