# Supplementary material for: The regulation loop of MARVELD1 interacting with PARP1 in DNA damage response maintains genome stability and promotes therapy resistance of cancer cells
Source: Cell Death Differ. 2023 Feb 7;30(4):922–37. doi: 10.1038/s41418-023-01118-z (PMC10070477; doi:10.1038/s41418-023-01118-z)
Supplement: Supplementary file 9 — Supplementary Table S1-6 [file 41418_2023_1118_MOESM9_ESM.docx]

**Table S1** Cell cycle analysis of HeLa/PC cells

| Phase of cell cycle | Ctrl | G1 | 0 h | 1 h | 2 h | 3 h | 4 h | 5 h | 6 h |
| --- | --- | --- | --- | --- | --- | --- | --- | --- | --- |
| G1 (%) | 71.7±3.5 | 100.0±0.0 | 100.0±0.0 | 100.0±0.0 | 100.0±0.0 | 100.0±0.0 | 100.0±0.0 | 100.0±0.0 | 100.0±0.0 |
| S (%) | 23.3±2.6 | 0.0±0.0 | 0.0±0.0 | 0.0±0.0 | 0.0±0.0 | 0.0±0.0 | 0.0±0.0 | 0.0±0.0 | 0.0±0.0 |
| G2 (%) | 4.9±0.9 | 0.0±0.0 | 0.0±0.0 | 0.0±0.0 | 0.0±0.0 | 0.0±0.0 | 0.0±0.0 | 0.0±0.0 | 0.0±0.0 |

**Table S2** Cell cycle analysis of HeLa/MARVELD1 cells

| Phase of cell cycle | Ctrl | G1 | 0 h | 1 h | 2 h | 3 h | 4 h | 5 h | 6 h |
| --- | --- | --- | --- | --- | --- | --- | --- | --- | --- |
| G1 (%) | 69.3±2.1 | 100.0±0.0 | 100.0±0.0 | 100.0±0.0 | 100.0±0.0 | 93.8±1.4 | 93.2±2.1 | 51.7±2.6 | 0.0±0.0 |
| S (%) | 25.9±1.3 | 0.0±0.0 | 0.0±0.0 | 0.0±0.0 | 0.0±0.0 | 6.3±1.3 | 6.7±2.1 | 48.3±2.4 | 100.0±0.0 |
| G2 (%) | 4.8±0.7 | 0.0±0.0 | 0.0±0.0 | 0.0±0.0 | 0.0±0.0 | 0.0±0.0 | 0.0±0.0 | 0.0±0.0 | 0.0±0.0 |

**Table S3** Primers used for the qPCR reaction

| Target | Species | Primers | Sequences (5′→3′) |
| --- | --- | --- | --- |
| MARVELD1 | Human | Forward | GCAACCTCAAGGATTACCCG |
|  |  | Reverse | CCATAGAGCGCCGAAAGCA |
| PARP1 | Human | Forward | CGGAGTCTTCGGATAAGCTCT |
|  |  | Reverse | TTTCCATCAAACATGGGCGAC |
| NAA50 | Human | Forward | TCCAGTCAGCTACAATGACAAGT |
|  |  | Reverse | CCTTCGGTAAGGTGCCAGAC |
| GAPDH | Human | Forward | AGGCTGTGGGCAAGGTCATC |
|  |  | Reverse | TCAGGTCCACCACTGACACG |
| MARVELD1 | Mouse | Forward | ACGTGCCGCCTGGAGAGCC |
|  |  | Reverse | GGCCATGAGGTCCACCACCTG |
| PARP1 | Mouse | Forward | GCTTTATCGAGTGGAGTACGC |
|  |  | Reverse | GGAGGGAGTCCTTGGGAATAC |
| GAPDH | Mouse | Forward | GCCTACCATGCCTTCCTG |
|  |  | Reverse | TCACACCACCTCCTCCTT |

**Table S4** Expression vectors used in this study

| Recombinant DNA | Source | Identifier |
| --- | --- | --- |
| pCDNA3.1-vector | Yu Li’s lab preserved | N/A |
| pCDNA3.1-MARVELD1-V5 | Yu Li’s lab preserved | N/A |
| pCDNA3.1-MARVELD1-Flag | This study | N/A |
| pCDNA3.1-MARVELD1-K53A-Flag | This study | N/A |
| pCDNA3.1-MARVELD1-K83A-Flag | This study | N/A |
| pCDNA3.1-MARVELD1-E85A-Flag | This study | N/A |
| pCDNA3.1-MARVELD1-D102A-Flag | This study | N/A |
| pCDNA3.1-MARVELD1-D118A-Flag | This study | N/A |
| pCDNA3.1-MARVELD1-K129A-Flag | This study | N/A |
| pCDNA3.1-MARVELD1-D130A-Flag | This study | N/A |
| pCDNA3.1-MARVELD1-K169A-Flag | This study | N/A |
| pCDNA3.1-MARVELD1-E171A-Flag | This study | N/A |
| pCDNA3.1-MARVELD1-3A-Flag | This study | N/A |
| pLVSIN-CMV-MARVELD1-EGFP | This study | N/A |
| pLVSIN-CMV-MARVELD1-3A-EGFP | This study | N/A |
| pRK5-HA-Ubiquitin | Yu Li’s lab preserved | N/A |
| pENTER-PARP1-Flag | Vigene Biosciences | Cat# CH870406 |
| pENTER-PARP1-1-331-Flag | This study | N/A |
| pENTER-PARP1-332-661-Flag | This study | N/A |
| pENTER-PARP1-662-1014-Flag | This study | N/A |
| pENTER-NAA50-Flag | Vigene Biosciences | Cat# CH812759 |
| pCMV-Myc-NAA50 | This study | N/A |

**Table S5** SiRNA target sequences

| Target | Primers | Sequences (5′→3′) |
| --- | --- | --- |
| siPARP1#458 | Forward | GCAAAGGCCAGGAUGGAAUTT |
|  | Reverse | AUUCCAUCCUGGCCUUUGCTT |
| siPARP1#1124 | Forward | GGACCAAGUGUAUGGUCAATT |
|  | Reverse | UUGACCAUACACUUGGUCCTT |
| siPARP1#1512 | Forward | GGAAGCCAACAUCCGAGUUTT |
|  | Reverse | AACUCGGAUGUUGGCUUCCTT |
| siNAA50#467 | Forward | GCUGGAGGUUGGCGAGCUATT |
|  | Reverse | UAGCUCGCCAACCUCCAGCTT |
| siNAA50#591 | Forward | GCACCUUACCGAAGGCUAGTT |
|  | Reverse | CUAGCCUUCGGUAAGGUGCTT |
| siNAA50#683 | Forward | GCAUGUCCAGAUCAGCAAUTT |
|  | Reverse | AUUGCUGAUCUGGACAUGCTT |
| siMARVELD1 | Forward | CCAGAAGCCUGGUUCCAAUTT |
|  | Reverse | AUUGGAACCAGGCUUCUGGTT |
| siNC | Forward | UUCUCCGAACGUGUCACGUTT |
|  | Reverse | ACGUGACACGUUCGGAGAATT |

**Table S6** Antibodies used in this study

| Antibodies | Source | Identifier |
| --- | --- | --- |
| Anti-MARVELD1 | Abcam | ab91640 |
| Anti-PARP1 | Cell Signaling | 9532s |
| Anti-PARP1 | Proteintech | 13371-1-AP |
| Anti-PARP1 | Proteintech | 66520-1-lg |
| Anti-NAA50 | Abclonal | A7387 |
| Anti-γ-H2AX | Cell Signaling | 80312 |
| Anti-8-OHdG | Trevigen | 4354-MC-050 |
| Anti-PAR | Trevigen | 4335-MC-100 |
| Anti-Ac-K | Cell Signaling | 9441s |
| Anti-ACTB | Abclonal | AC026 |
| Anti-H3.1 | Proteintech | 17168-1-AP |
| Anti-GAPDH | Sungene | KM9002 |
| Anti-Flag-Tag | Cell Signaling | 14793 |
| Anti-V5-Tag | Cell Signaling | 13202 |
| Anti-HA-Tag | Proteintech | 51064-2-AP |
| Anti-MYC-Tag | Proteintech | 16286-1-AP |
| Anti-AIMP2 | Proteintech | 10424-1-AP |
| Anti-FUBP1 | Proteintech | 24864-1-AP |
| Anti-KAP1 | Proteintech | 15202-1-AP |
| Anti-PRDX1 | Proteintech | 15816-1-AP |
| Anti-PRDX3 | Proteintech | 10664-1-AP |
| Anti-PARK7 | Proteintech | 11681-1-AP |
| Anti-ARF4 | Proteintech | 11673-1-AP |
| Anti-CCT1(TCP1) | Proteintech | 10320-1-AP |
| Anti-CCT2 | Proteintech | 24896-1-AP |
| Anti-CCT3 | Proteintech | 10571-1-AP |
| Anti-CCT6-A | Proteintech | 19793-1-AP |
| Anti-MCM3 | Proteintech | 15597-1-AP |
| Anti-MCM4 | Proteintech | 13043-1-AP |
| Anti-MCM7 | Proteintech | 11225-1-AP |
| Anti-YWHAE | Proteintech | 11648-2-AP |
| Anti-YWHAG | Proteintech | 12381-1-AP |
| Anti-YWHAZ | Proteintech | 14881-1-AP |
| Anti-DDB1 | Proteintech | 11380-1-AP |
| Anti-PABPC1 | Proteintech | 10970-1-AP |
| Anti-XRCC5 | Proteintech | 16389-1-AP |
| Anti-Goat anti-mouse IgG, HRP conjugate | Proteintech | SA00001-1 |
| Anti-Goat anti-rabbit IgG, HRP conjugate | Proteintech | SA00001-2 |
| Anti-Goat anti-rabbit IgG, Alexa Fluor488 | Invitrogen | A-11034 |
| Anti-Goat anti-mouse IgG, Alexa Fluor594 | Invitrogen | A-11005 |
